# *Trichuris trichiura* (Linnaeus, 1771) From Human and Non-human Primates: Morphology, Biometry, Host Specificity, Molecular Characterization, and Phylogeny

**DOI:** 10.3389/fvets.2020.626120

**Published:** 2021-02-09

**Authors:** Julia Rivero, Cristina Cutillas, Rocío Callejón

**Affiliations:** Department of Microbiology and Parasitology, Faculty of Pharmacy, University of Seville, Seville, Spain

**Keywords:** *Trichuris trichiura*, non-human primates, ribosomal DNA, mitochondrial DNA, zoonoses

## Abstract

Human trichuriasis is a Neglected Tropical Disease, which affects hundreds of millions of persons worldwide. Several studies have reported that non-human primates (NHP) represent important reservoirs for several known zoonotic infectious diseases. In this context, *Trichuris* infections have been found in a range of NHP species living in natural habitats, including colobus monkeys, macaques, baboons, and chimpanzees. To date, the systematics of the genus *Trichuris* parasitizing humans and NHP is unclear. During many years, *Trichuris trichiura* was considered as the whipworm present in humans and primates. Subsequently, molecular studies suggested that *Trichuris* spp. in humans and NHP represent several species that differ in host specificity. This work examines the current knowledge of *T. trichiura* and its relationship to whipworm parasites in other primate host species. A phylogenetic hypothesis, based on three mitochondrial genes (*cytochrome c oxidase* subunit 1, *cytochrome* b, and large subunit rRNA-encoding gene) and two fragments of ribosomal DNA (Internal Transcribed Spacer 1 and 2), allowed us to define a complex of populations of *T. trichiura* hosting in a large variety of NHP species, in addition to humans. These populations were divided into four phylogenetic groups with a different degree of host specificity. From these data, we carry out a new morphological and biometrical description of the populations of *Trichuris* based on data cited by other authors as well as those provided in this study. The presence of *T. trichiura* is analyzed in several NHP species in captivity from different garden zoos as possible reservoir of trichuriasis for humans. This study contributes to clarify questions that lead to identification of new taxa and will determine parasite transmission routes between these primates, allowing the implementation of appropriate control and prevention measures.

## Introduction

Worldwide, ~1.5 billion people, nearly 24% of the world's population, are infected with soil-borne helminths. Soil-borne helminthiasis is widely distributed in tropical and subtropical areas, especially in sub-Saharan Africa, The Americas, China, and East Asia. More than 267 million preschoolers and more than 568 million school-age children live in areas with severe transmission of these parasites and need treatment and preventive interventions. The main species of soil-borne helminths that infect humans are *Ascaris lumbricoides* (roundworm), *Trichuris trichiura* (whipworm), and *Necator americanus* and *Ancylostoma duodenale* (hookworms) (1).

*T. trichiura*, is the etiological agent of the parasitic disease known as “trichuriasis,” which is considered as a Neglected Tropical Disease. *T. trichiura* is the second most common helminth in humans, and Trichuriasis has a worldwide geographical distribution. The prevalence is higher in places with warm and humid weather, where there is a lack of basic sanitation services. Between 30 and 80% of cases are recorded in children, who suffer the greatest parasitic burden and those with the most significant symptoms (2). Transmission of this parasite occurs after ingestion of embryonated eggs. These eggs can enter new hosts through contaminated hands, food, soil, and water. Then these hatch in the intestine, where L1 larvae are released. Larvae penetrate the epithelial layer of the large intestine and grow to adult stage. After mating, the non-embryonated eggs are released from the females and again reach the environment through the host's feces.

Up to date, whipworms isolated from humans and other primates have traditionally been regarded as *T. trichiura* (3–5), while those recovered from pigs and wild boars are known as *Trichuris suis* (6, 7). It is well-known that differentiation between closely related species of *Trichuris* is very difficult due to the phenotypic plasticity of the organisms themselves; host-induced variation, paucity of morphological features, and overlapping morphological characteristics that occur among species (8–11). Thus, many studies on *Trichuris* have focused on the morphological and molecular differentiation of *T. trichiura* and *T. suis*, which are molecularly different but morphologically similar (7, 12–14).

On the other hand, the relationship between *Trichuris* from humans and non-human primates (NHP) in terms of genetic and evolutionary aspects is poorly understood. In recent years, some publications addressed the question of whether *Trichuris* species are shared between humans and NHP or whether there are different species. The genus *Trichuris* is likely a candidate to harbor cryptic species as it has a wide geographical distribution and infects several host species (15). As revealed by recent studies, there is more than one taxon capable of infecting humans and other primates, including individuals in captivity, suggesting that *T. trichiura* should be considered a complex species that includes different cryptic units (16, 17). In addition, based on morpho-biometric and molecular parameters, new species of *Trichuris* have been described in primates, such as *Trichuris rhinopiptheroxella* (18) that was found in the golden snub-nosed monkey (*Rhinopithecus roxellana*), *Trichuris colobae* from *Colobus guereza kikuyuensis* (19), and *Trichuris ursinus* from *Papio ursinus* (20). Therefore, these studies confirmed that *T. trichiura* is not the only whipworm found in primates.

Currently, the systematics of the genus *Trichuris* shows significant gaps. This is due to two main reasons: (i) the lack of comparative morpho-biometric data through the use of multiple parameters and statistical tests applied to the taxonomic study of these species and (ii) the paucity of published research on the genetics of the different *Trichuris* species in humans, NHP, and pigs. Nowadays, researchers have not yet managed to finally establish the degree of divergence between the different genetic lineages that appear to exist in *Trichuris* species parasitizing these hosts.

In this paper, we carried out an update of the morphological and biometric characteristics of *T. trichiura* isolated from human and NHP. Besides, the molecular phylogenetic relationships between these populations are analyzed based on molecular data (mitochondrial and nuclear markers). Furthermore, some phylogenetic hypotheses were inferred for *Trichuris* spp. to shed light on the degree of divergence between different genetic lineages. In addition, the presence of *T. trichiura* was analyzed in several NHP species in captivity from different garden zoos as possible reservoir of trichuriasis for humans.

## Materials and Methods

### Ethics Statement

This study does not require approval by an ethics committee. *Macaca sylvanus* and *C. g. kikuyensis*, from which *Trichuris* specimens were collected from their caeca *postmortem*, died of natural death. The specimens were handled and housed in a zoo in strict accordance with good animal practices. The other specimens and eggs of *Trichuris* sp. were recovered from the feces after routine anthelmintic treatment.

### Isolation of Material

In this study, we sampled *Trichuris*'s adults and eggs of a total of five NHP host species, including the Barbary macaque (*M. sylvanus*), vervet monkey (*Chlorocebus aethiops*), patas monkey (*Erythrocebus patas*), Guinea baboon (*Papio papio*), and black and white colobus (*C. g. kikuyuensis*) from the Zoo Castellar (Cádiz, Spain), Selwo Aventura (Málaga, Spain), Zoo Barcelona (Barcelona, Spain), Parque de la Naturaleza de Cabárceno (Cantabria, Spain), and Bioparc Fuengirola (Málaga, Spain), respectively (Table 1).

**Table 1:** DNA obtained from the samples.

**Zoo gardens**	**Hosts species**		**N° adults/eggs analyzed**	***Trichuris* species**	**Haplotypes obtained**					**Number of base pairs/G+C**				
	**Scientific name**	**Common name**			***cox*1**	***co*b**	***rrn*L**	**ITS1**	**ITS2**	***cox*1**	***co*b**	***rrn*L**	**ITS1**	**ITS2**
Zoo Castellar	*Macaca sylvanus*	Barbary macaque	43 adults	*T. trichiura*	4 H (^*^)	5 H (^*^)	5 H	6 H	8 H (^*^)	370/38–39.2	520/30.2–31	386–387/29.7–31.3	586–597/63.1–64.3	514–587/63.7–65.2
Selwo Aventura	*Chlorocebus aethiops*	Vervet monkey	5 sample batches of eggs (40–65 eggs/batch)		1 H	1 H	-	2 H	5 H	370/38.6	522/30.1	–	594–601/61.4–62.3	556–580/62.1–64.1
Zoo Barcelona	*Erythrocebus patas*	Patas monkey	1 sample batch of eggs		1 H	1 H	-	1 H	1 H	332/38	520/29.6	–	593/64.1	436/65.6
Parque de la Naturaleza de Cabarceno	*Papio papio*	Guinea baboon	3 adults and 9 sample batches of eggs (40–200 eggs/batch)		1 H	4 H	1 H	2 H	5 H	370/38.6	520/29.6–30.8	387/30	588–607/61.4–64.3	602–611/62.8–63.2
Bioparc Fuengirola	*Colobus guereza kikuyuensis*	Black and white colobus	5 adults	*T. colobae*	-	-	5 H	-	-	-	-	395/30.1–30.9	-	-

H, haplotype;

(*) *analyzed by Rivero et al. (17)*.

Only three adult whipworm specimens (two females and one male) were collected from Guinea baboon, sixty-five adults (32 females and 33 males) from a male Barbary macaque (17, 21) and five adults from a *C. g. kikuyuensis*. Adult's worms were washed separately in saline solution (0.9% w/v), then frozen at −20°C until posterior studies. Whipworm's eggs were isolated from feces of all NHP species. The sequences successfully obtained of different molecular markers are summarized in the Table 1.

The fecal eggs were concentrated using a Sheather's sugar solution (22) and then embryonated at 32°C for 3–4 weeks added with potassium dichromate 0.2% w/v solution to give humidity to the medium and prevent fungal and bacterial growth (23). Subsequently, the worms were measured and genomic DNA, from both worms and eggs, was extracted.

### Morphological Study

Three adult whipworms from Guinea baboon were identified according to previous studies (7, 19, 20). We carried out morphological studies as described Oliveros et al. (24) and Skrjabin et al. (25). *Trichuris* specimens were measured according to parameters reported by Spakulová and Lýsek (26), Suriano and Navone (27), and Robles et al. (28), and a comparative study was carried out with *T. trichiura*'s specimens previously analyzed biometrically and molecularly (7, 14, 17, 21).

### Molecular Study

#### PCR and Sequencing of Specimens

Genomic DNA from adult worms and a pack of 45–200 isolated eggs were extracted using DNeasy Blood and Tissue Kit (Qiagen) according to the manufacturer's protocol. Quality of extractions was assessed using 0.8% agarose gel electrophoresis infused with SYBR^®^ Safe DNA gel stain.

All molecular markers sequenced in the present study [*cox*1, *co*b and *rrn*L mitochondrial DNA (mtDNA) and ITS1 and ITS2 ribosomal DNA (rDNA)] were amplified using the polymerase chain reaction (PCR) by a thermal cycler (Eppendorf AG; Hamburg, Germany). PCR mix, PCR conditions, and PCR primers are summarized in the Supplementary Table 1.

The PCR products were checked on SYBR^®^ Safe stained with 2% w/v Tris–borate–EDTA (TBE) agarose gels. Bands were eluted and purified from the agarose gel using the QWizard SV Gel and PCR Clean-Up System Kit (Promega, Madison, WI, U.S.A.). Once purified and concentrated, the products were sequenced by Stab Vida (Lisbon, Portugal).

#### Phylogenetic Analysis

To assess the similarity among all marker sequences of *Trichuris* sp. obtained in the present study and other *Trichuris* species, the number of nucleotide differences per sequence was analyzed using Compute Pairwise Distances based on the number of differences method of MEGA v7.0 (29).

To obtain a nucleotide sequence alignment file, the MUSCLE alignment method was used in MEGA v7.0 (29). Additional sequences from the National Center for Biotechnology Information (NCBI) GenBank^®^ database were incorporated into the alignments (Supplementary Table 2).

Assessment of nucleotide substitution saturation, an indicator of whether the genetic marker is useful, was performed using DAMBE v5.3.32 (30, 31). Saturation was based on the values of Iss (index of substitution saturation) and Iss.c (critical Iss value), where Iss < Iss.c indicated that the genetic marker was not saturated and vice versa. Haplotype diversity (h) and nucleotide diversity (π) were calculated using DnaSP v6.12.03 (32).

All phylogenetic trees were inferred based on nucleotide data and obtained by two methods: Maximum Likelihood (ML) and Bayesian Inference (BI). PHYML package was used to generate ML trees (33), and MrBayes v3.2.6 to generate BI (34). jModelTest was employed to resolve the best-fit substitution model for the parasite data (35). Models of evolution were selected for subsequent analysis according to the Akaike Information Criterion (36). To examine the dataset containing the concatenation of four markers used (ITS1, ITS2, *cox*1, and *co*b), analyses based on BI were partitioned by gene and models for individual genes within partitions were those selected by jModelTest. For ML inference, best-fit nucleotide substitution models included the general time-reversible (GTR) model with gamma-distributed rate variation (G) and a proportion of invariable sites (I), GTR + G (ITS1 and ITS2), GTR + I + G (*cox1*), GTR + I + G (*co*b), and GTR + G (*rrn*L). Support for the topology was examined using bootstrapping (heuristic option) (37) over 1,000 replications to assess the relative reliability of clades. The commands used in MrBayes for BI were *nst* = 6 with gamma rates (ITS1, ITS2, and *rrn*L), *nst* = 6 with invgamma rates (*cox*1 and *co*b), and *nst* = mixed (concatenated phylogenetic trees). The standard deviation of split frequencies was used to determine whether the number of generations completed was sufficient; the chain was sampled every 500 generations, and each dataset was run for 10 million generations. Trees from the first million generations were discarded based on an assessment of convergence. Burn-in was determined empirically by examination of the log likelihood values of the chains. The Bayesian Posterior Probabilities (BPP) comprise the percentage converted.

## Results

### Molecular Analysis

#### Annotation and Features of Ribosomal and Mitochondrial Genomes

The successfully sequenced specimens, the length of the different sequences, the content of G + C%, and haplotypes of ribosomal and mitochondrial markers analyzed at the present study are shown in Table 1. Different repetitive nucleotide sequences, termed ≪ microsatellites ≫, were found in the ITS2 sequences of *Trichuris* sp. from human and different NHP. Thus, Poly (GCA), Poly (CGA), and Poly (GCG) were observed in positions 280, 302, and 344, respectively. Furthermore, Poly (GCA) and Poly (GGC) were found in the ITS1 sequences in positions 77 and 203, respectively. Also, a common area to all species of *Trichuris* was observed in positions 280 (GATCTGGGTGT) and 286 (GCCGCCGGTT) in this ITS1 sequence.

Nucleotide sequence data reported in this study were deposited at the GenBank™, EMBL, and DDBJ databases, and the accession numbers are available in Supplementary Table 2.

#### Phylogenetic Analysis

**Figure 1:**
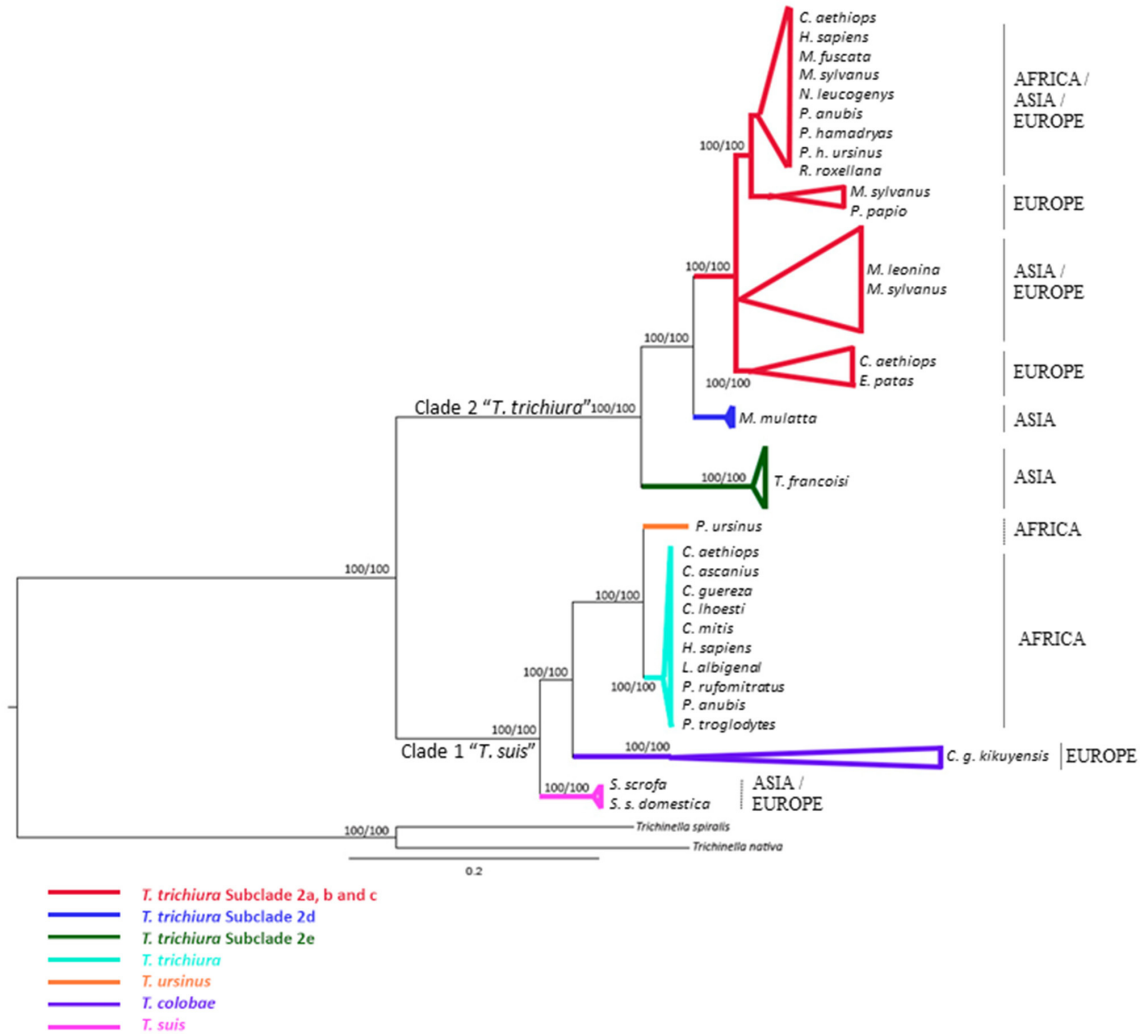
Phylogenetic tree of *Trichuris* species based on combined analysis of ribosomal DNA (ITS1 and ITS2) inferred using Bayesian Inference. Maximum Likelihood bootstrap values of clades are listed first, followed by Bayesian Posterior Probabilities, respectively, for clade frequencies exceeding 60%.

**Figure 2:**
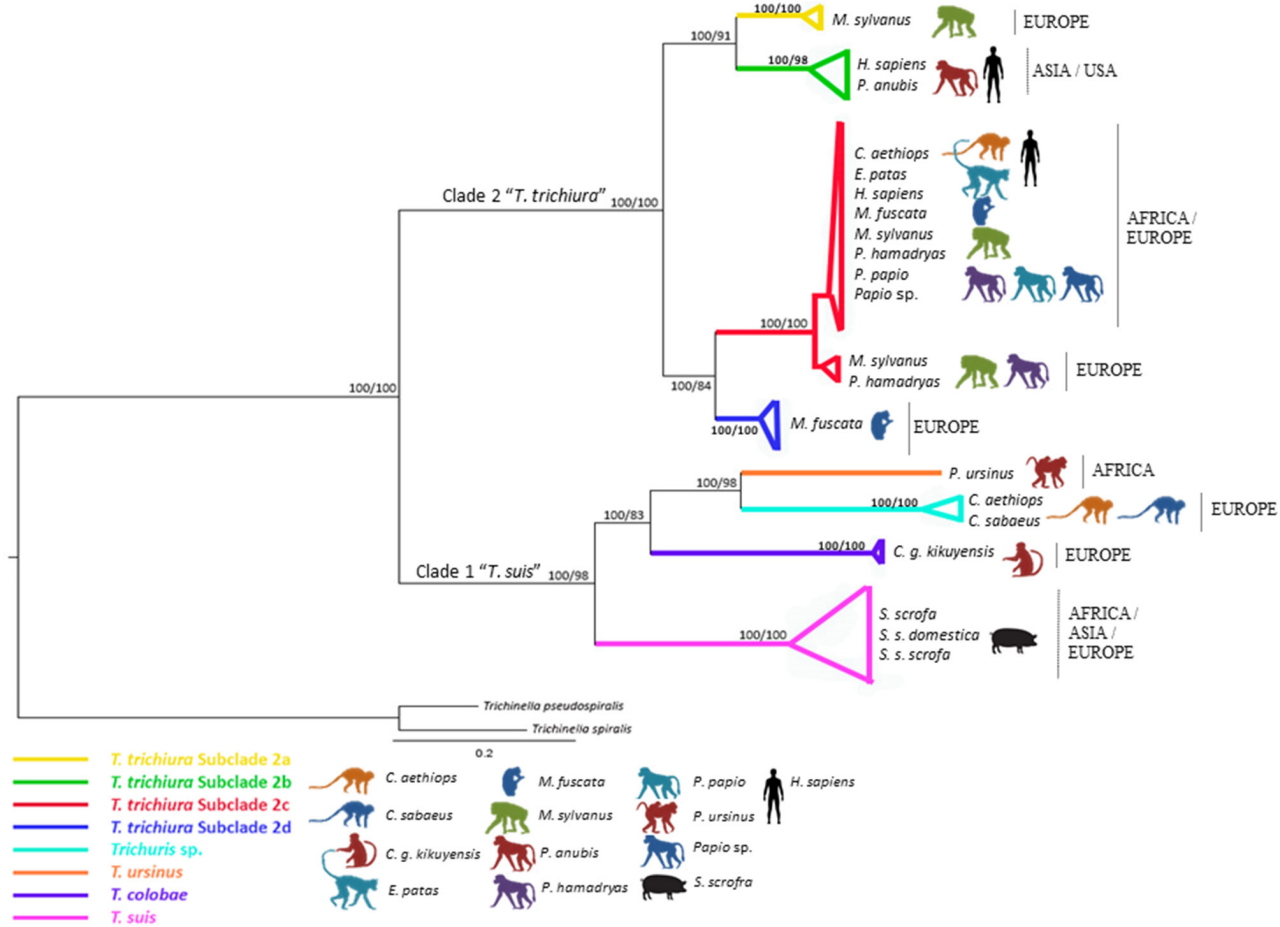
Phylogenetic tree of *Trichuris* species based on combined analysis of mitochondrial DNA (*cox*1 and *co*b) inferred using Bayesian Inference. Maximum Likelihood bootstrap values of clades are listed first, followed by Bayesian Posterior Probabilities, respectively, for clade frequencies exceeding 60%.

**Figure 3:**
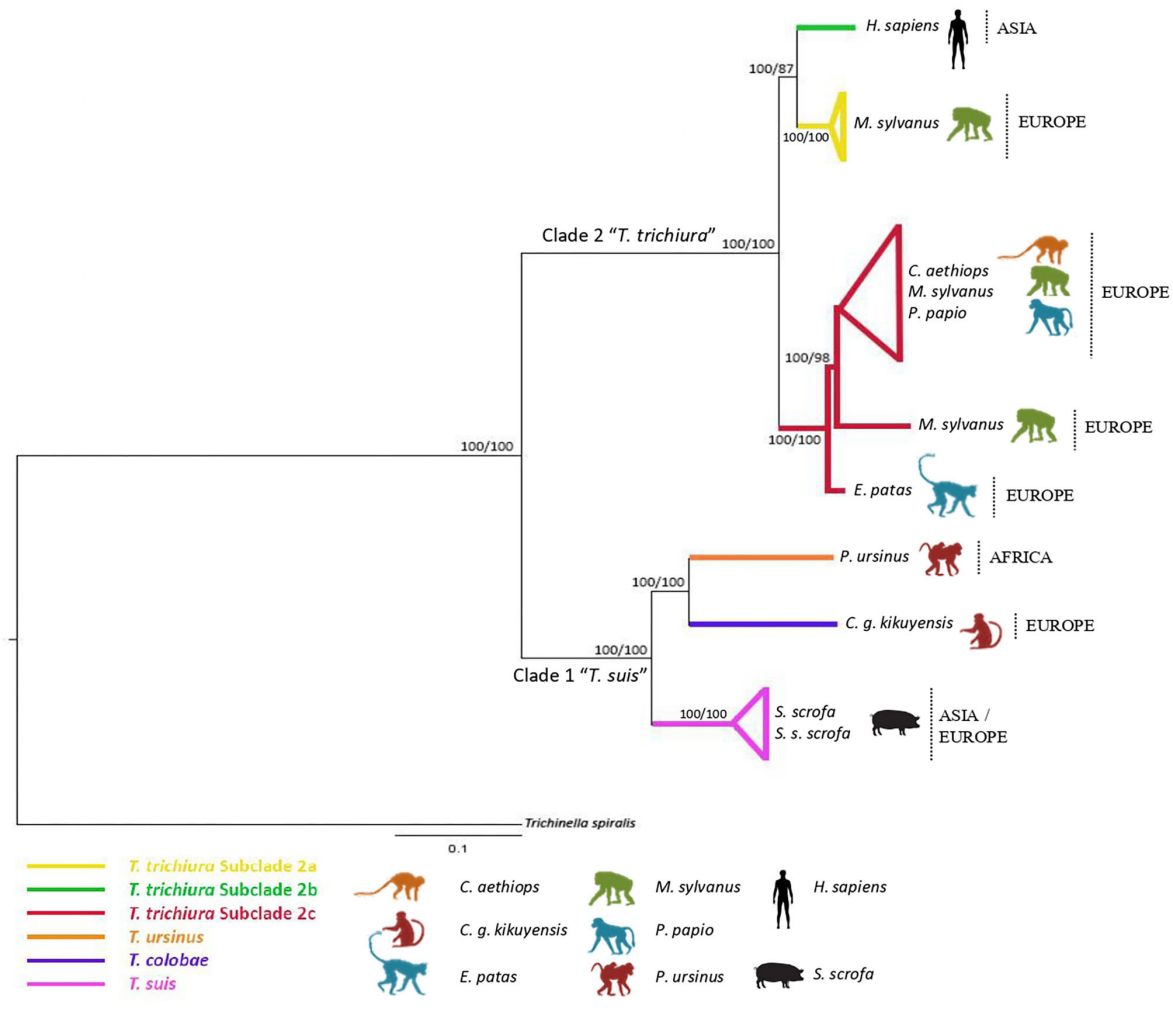
Phylogenetic tree of *Trichuris* species based on combined analysis of mitochondrial DNA (*cox*1, *co*b) and nuclear ribosomal DNA (ITS1 and ITS2) inferred using Bayesian Inference. Maximum Likelihood bootstrap values of clades are listed first, followed by Bayesian Posterior Probabilities, respectively, for clade frequencies exceeding 60%.

All phylogenetic trees based on ribosomal and mitochondrial markers (partitioned and concatenated) confirmed the existence of two main clades: clade 1: “*Trichuris suis*” and clade 2: “*Trichuris trichiura*” (Figures 1–**3** and Supplementary Figures 1–3).

The alignment of 46 ITS1 and 79 ITS2 rDNA sequences of *Trichuris* species yielded a dataset of 876 and 863 characters, respectively. Based on ITS1 and ITS2 sequences, the concatenated phylogenetic tree revealed the existence of two highly supported phylogenetic groups within clade 2 “*T. trichiura*”: One group corresponded to *T. trichiura* lineage (100% ML and 100% BPP) (Supplementary Table 3) clustering *Trichuris* sp. from different hosts and geographical regions in six subclades (100% ML and 100% BPP) that included *Trichuris* sp. from *Trachypithecus francoisi* (Figure 1). The other 5 subclades (except *Trichuris* sp. from *T. francoisi*) showed a high homology each other, ranging 94.65–99.65% (Supplementary Table 4).

The phylogeny inferred on mitochondrial datasets (partitioned and concatenated) revealed similar topologies (Figure 2 and Supplementary Figures 1–3). Thus, four main clades were observed in “*T. trichiura* lineage” where *Trichuris* sp. from *P. papio, C. aethiops*, and *E. patas* clustered together in the subclade named 2c with *T. trichiura* from *Homo sapiens* from Uganda and *Trichuris* sp. from other hosts from Africa and Europe (Figure 2 and Supplementary Figures 1, 2).

The multiple alignments of 48 *cox*1 nucleotide sequences (including outgroups) yielded a dataset of 296 characters. The phylogenetic tree based on *cox*1 showed *Trichuris* from *E. patas, C. aethiops*, and *P. papio* clustering in subclade 2c (European and African origin) and related with *Macaca fuscata* (subclade 2d). This marker did not resolve subclade 2a (Asian and USA origin) appearing in polytomy (Supplementary Figure 1 and Supplementary Table 3).

The multiple alignments of 47 *co*b nucleotide sequences (including outgroups) yielded a dataset of 444 characters. The phylogenetic tree based on *co*b was in congruence with *rrn*L phylogenetic inferences of *T. trichiura* lineage. Nevertheless, the sister relationship between subclades 2a, 2b, and 2d was not supported (Supplementary Figure 2 and Supplementary Table 3).

The *rrn*L dataset included 358 aligned positions and 76 taxa, including outgroups. ML and BI methods showed congruence between each other revealing two main clades (“*T. suis* lineage” and “*T. trichiura* lineage”) and respect to the sister-group relationships between *Trichuris* spp. from NHP, humans and pigs (Supplementary Figure 3 and Supplementary Table 3). Within clade 2 “*T. trichiura* lineage,” phylogenetic trees confirmed the existence of four different subclades highly supported clustering subclade 2b: *T. trichiura* from *H. sapiens* from China and *Papio anubis* from the USA and subclade 2a including the minority haplotype of *T. trichiura* from *M. sylvanus* from Spain; subclade 2c: *T. trichiura* from *P. papio, Chlorocebus sabaeus* and *M. sylvanus* from Spain, *H. sapiens* from Uganda, *Papio hamadryas* from Europe, and two haplotypes of *Trichuris* sp. from *M. fuscata* from Europe; and subclade 2d: *T. trichiura* from *M. fuscata* from Europe (Supplementary Figure 3). Curiously, the minority haplotype of *M. sylvanus* (subclade 2a) and *M. fuscata* (subclade 2d) (European origin) appeared related with those from Asia and USA (subclade 2b) (Supplementary Figure 3 and Supplementary Table 3). In addition, a sister relationship between *T. trichiura* and *Trichuris* sp. from *T. francoisi* was observed, both species within “*T. trichiura* lineage” (clade 2).

The concatenated dataset of mitochondrial gene (*cox*1 and *co*b) sequences (Figure 2) revealed the subclade 2c as the main one clustering the majority of *T. trichiura* parasitizing African humans and different NHP from Africa and Europe, showing a sister relationship between 2c and 2d and besides between 2a and 2b (Asian and USA origins) (Figure 2).

The concatenated dataset of ribosomal (ITS1 and ITS2) and mitochondrial (*cox*1 and *co*b) gene sequences included 2,479 aligned sites and only 13 taxa, since only we could concatenate sequences of the same individual. Phylogenetic analyses of this dataset yielded a tree with branches strongly supported. Thus, the *T. trichiura* population was separated in only three different subclades, the subclade 2d not being represented due to the absence of sequences in all the markers. Subclade 2c was the most representative subclade including populations from a high variety of host species (Figure 3 and Supplementary Table 3).

The phylogenetic inferences revealed that the populations of *Trichuris* from *P. papio, C. aethiops*, and *E. patas* analyzed in the present study clustered mainly in the subclade of *T. trichiura* (subclade 2c).

#### Comparative Sequence Analysis

In order to analyze the intraspecific and interspecific similarity in *T. trichiura* and between *T. trichiura* and *Trichuris* spp. parasitizing NHP as well as *T. suis*, we carried out a comparative study considering the different clades and subclades previously described for *Trichuris* spp. hosting humans, NHP, and swine (Supplementary Tables 4–7).

Thus, by examining ITS1 and ITS2 sequences, specimens obtained from *P. papio, C. aethiops*, and *E. patas* from Spain revealed a high similarity with populations of *T. trichiura* corresponding to subclades 2a, 2b, and 2c and subclade *Macaca mulatta* from different geographical origins (94.65 to 100%) (Supplementary Table 4). Further, the similarity was lower when clade 2 (*T. trichiura* lineage) and clade 1 (*T. suis*) were compared (90.62–92.23%).

Mitochondrial sequences (*cox*1 and *co*b) from *T. trichiura* from *P. papio, C. aethiops*, and *E. patas* from Spain showed the highest intraspecific similarity within subclade 2c (97.3 to 100% and 92.12 to 100%, respectively) (Supplementary Tables 5, 6). For *rrn*L sequences, similar results were observed for *T. trichiura* from *M. sylvanus* and *P. papio* (97.77–100%). However, based on three mitochondrial markers, *T. trichiura* from *M. sylvanus* showed two different lineages corresponding to subclades 2a and 2c (Supplementary Table 7). Based on three mitochondrial markers, the similarity between clade 2 and *T. suis* ranged from 68.47 to 82.68%, values lower than those shown within clade 2.

Analysis of genetic diversity for clade 2 based on ITS and mitochondrial sequences revealed a haplotype diversity of 1.0 and 0.95–0.93, respectively (Table 2). In addition, nucleotide diversity based on ITS and *rrn*L sequences was 0.05; nevertheless, mitochondrial genes (*cox*1 and *co*b genes) revealed a higher nucleotide diversity (0.09–0.11) with the maximum values for *cox*1 (0.11) (Table 2). Within clade 2 (subclades 2a, 2b, 2c, and 2d), haplotype diversity of mitochondrial genes ranged 0.71–1.0 for *cox*1, 0.67–1.0 for *co*b, and 0.88–1.0 for *rrn*L with the maximum values for subclade 2a. Nucleotide diversity of mitochondrial genes ranged 0.01–0.04 for *cox*1 and *co*b with the maximum values for subclade 2b, and the value for *rrn*L was 0.01 (Table 2).

**Table 2:** Summary of genetic measures for *cox*1, *cob, rrn*L genes, and ITS region sequences.

	**Subclade 2a**			**Subclade 2b**			**Subclade 2c**			**Subclade 2d**			***Trichuris trichiura*** **complex (Clade 2)**			
	***cox*1**	***co*b**	***rrn*L**	***cox*1**	***co*b**	***rrn*L**	***cox*1**	***co*b**	***rrn*L**	***cox*1**	***co*b**	***rrn*L**	***cox*1**	***co*b**	***rrn*L**	**ITS**
Number of haplotypes/number of sequences	3/3	3/3	2/2	3/4	2/3	10/13	5/13	14/18	15/29	6/6	4/5	5/6	17/26	23/29	31/50	21/22
Haplotype diversity	1.0	1.0	1.0	0.83	0.67	0.95	0.71	0.95	0.88	1.0	0.90	0.93	0.93	0.98	0.95	1.0
Nucleotide diversity	0.02	0.02	0.01	0.04	0.04	0.01	0.01	0.02	0.01	0.02	0.01	0.01	0.11	0.09	0.05	0.05
Nucleotide saturation^*^	-	-	-	-	-	-	-	-	-	-	-	-	No	No	No	Yes

* *Substitution saturation test for partial cox1, cob, rrnL genes, and ITS sequences of Trichuris trichiura (Clade 2). “Yes” indicates that most of this sites have already been changed before (Iss>Iss.c), indicating nucleotide saturation*.

The analysis of nucleotide substitution saturation based on nuclear markers showed Iss > Iss.c (*P* < 0.005), indicating that ITS regions were saturated. Nevertheless, mitochondrial genes (*cox*1, *co*b, and *rrn*L) were not saturated (Iss < Iss.c, *P* < 0.005). Genetic diversity measures for all markers are summarized in Table 2.

### Update Morphological Description of *T. trichiura* (Linnaeus, 1771)

Taxonomic summary

- Class Enoplea (Order Trichocephalida) (12, 38).
- Host: *H. sapiens* (14), *Pan troglodytes* (7), *M. sylvanus* (17, 21), *P. papio* (present study).

**Figure 4:**
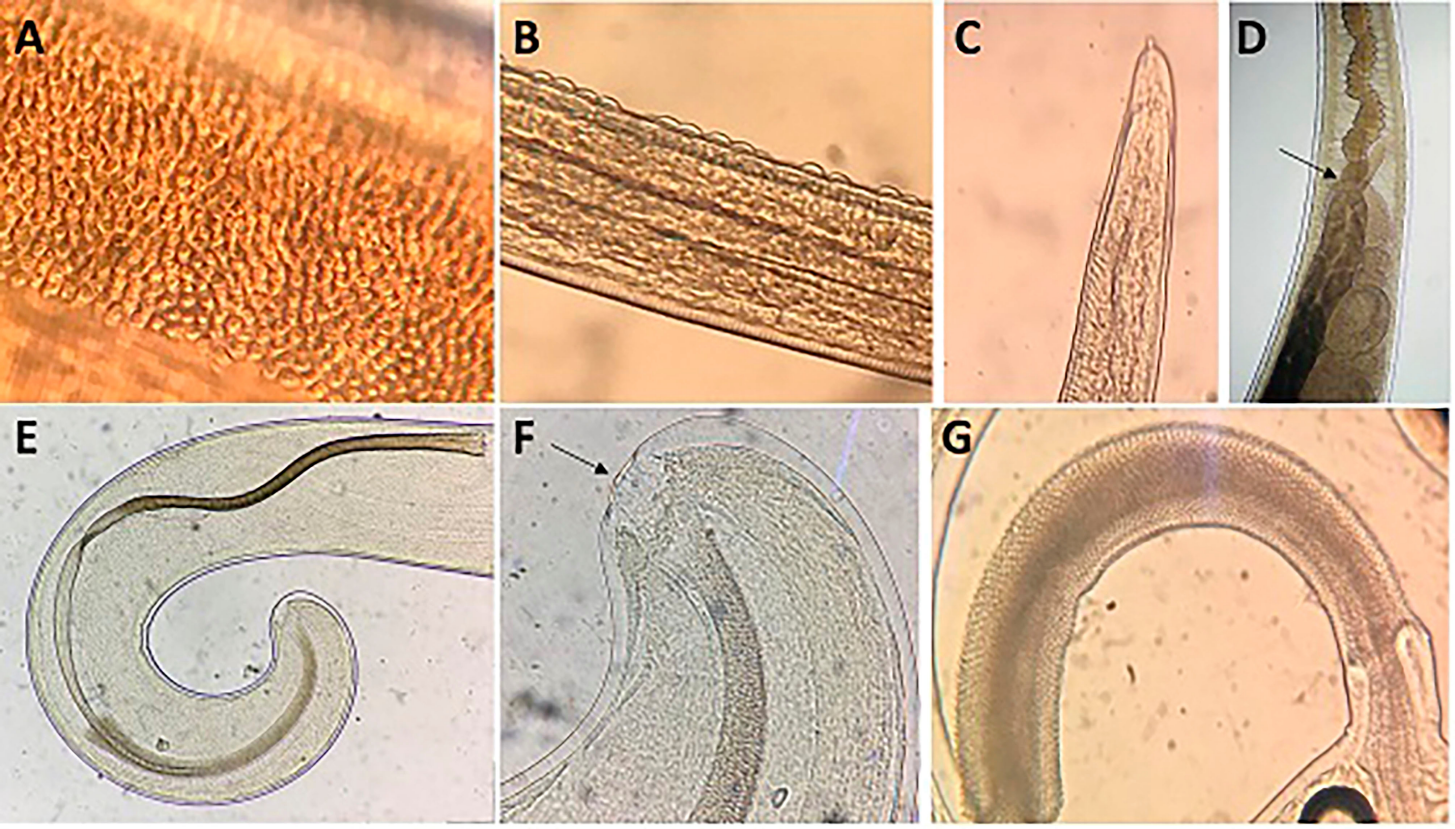
Morphology of males of *T. trichiura*. **(A)** Tuberculate band. **(B)** Bacillary band showing typical cuticular inflations. **(C)** The mouth with the stylet. **(D)** Stichosome with 1 row of stichocytes and 1 pair of conspicuous cells at esophagus–intestinal junction level (arrowed). **(E)** Spicule. **(F)** Papillae pericloacal. **(G)** Spicule sheath.

**Figure 5:**
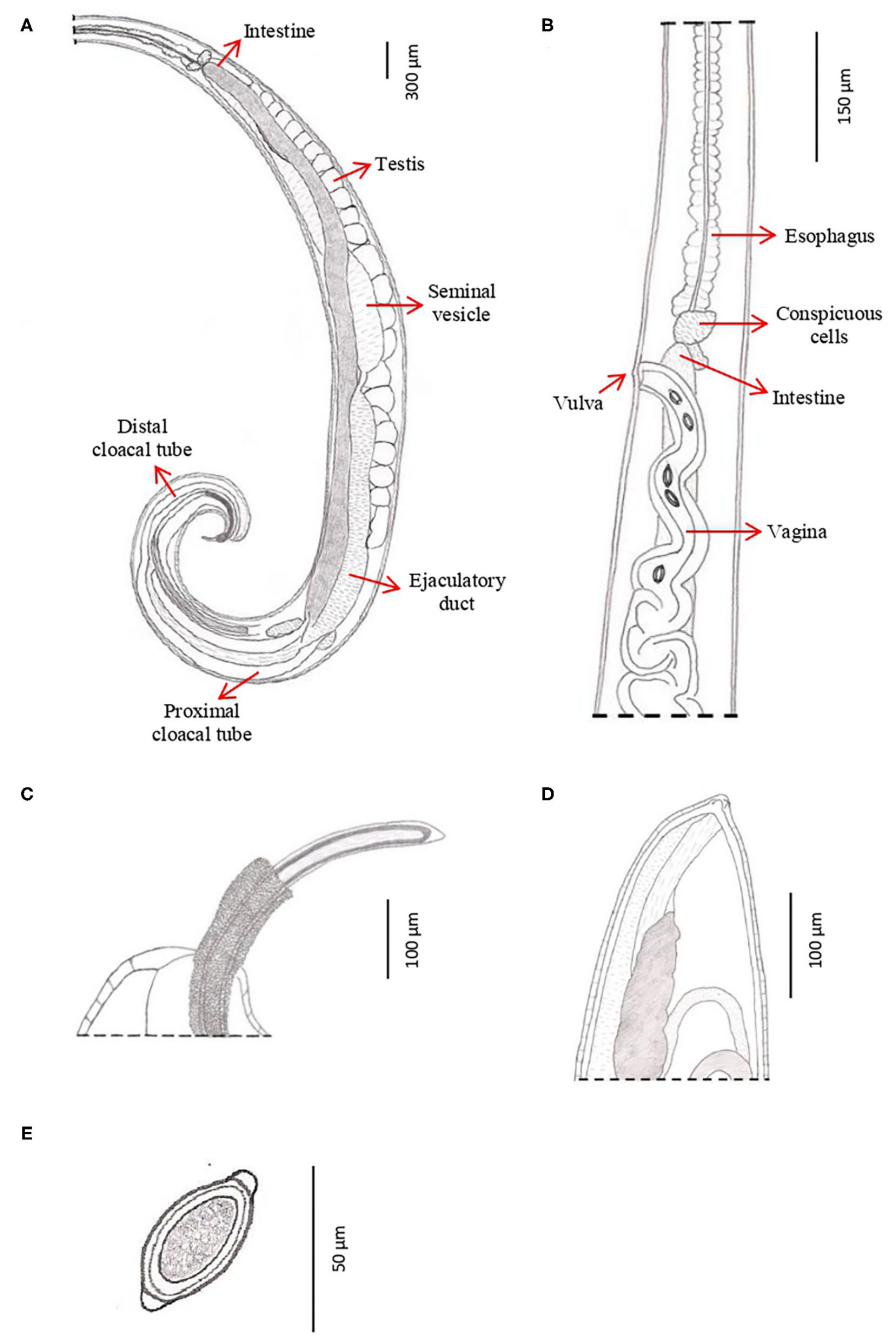
Drawings of *T. trichiura* from *P. papio*. **(A)** Male, posterior end, spiny spicule sheath, spicule, spicule tube and proximal and distal cloacal tube, lateral view. **(B)** Female, esophagus–intestine junction, vulva and vagina, lateral view. **(C)** Male, detail of the posterior end, lateral view. **(D)** Female, posterior end, lateral view. **(E)** Eggs.

*General:* This is a parasite with a filiform anterior part and a broad and handle-like posterior part. The thin anterior portion of the parasite displays 2 types of cuticle patterns. One side is distinctly striated with transverse grooves and the other side in a finely tuberculate band under higher magnification reveals small circular elevated bodies, which are evenly distributed (Figure 4A). The ventral side of the anterior part of the body is slightly tapered toward the cephalic end which presents a broad longitudinal elongated “bacillary band” showing typical cuticle inflations (Figure 4B). The mouth is surrounded by a group of cephalic papillae arranged in two circles (an inner circle and a lateral circle) with a conspicuous organ, the stylet (Figure 4C), protruding from the middle portion of the mouth cavity. Adults present a moniliform esophagus constituted with a short muscular zone and a long stichosome with 1 row of stichocytes (Figure 4D), and 1 pair of conspicuous cells at esophagus-intestinal junction level (Figures 4D, 5A,B). The site where the esophagus transforms into the intestine corresponds to the place of transition of the thin anterior part into the thick posterior and shows typical glandular cells at this point (Figures 4D, 5A,B).

*Male:* The body is 25–49.3 mm long. The ratio between anterior and posterior body length is 2.6–4:1. The thin anterior part is 1:1.4–1.7 of the entire length of the body. The length of the esophagus is 18–28.9 mm. The width of the body in the esophageal end is 0.15–0.49 mm, and the maximum width (in the posterior part of the body) is 0.39–0.74 mm (Table 3). Posterior end of the body ventrally incurved (Figures 4E–G).

**Table 3:** Biometrical data of males of *T. trichiura*.

	***T. trichiura* (Present study, updated)**	***T. trichiura*** **from** ***P. troglodytes*** **[Cutillas et al.**, **(****7****)]**				***T. trichiura*** **from** ***Homo sapiens*** **[Nissen et al**. **(****14****)]**			***T. trichiura*** **from** ***M. sylvanus*** **[Garc**í**a-Sánchez et al**. **(****21****)]**				***T. trichiura*** **from** ***P. papio*** **(Present study)**		
	**MIN-MAX**	**MIN**	**MAX**	**X**	**δ**	**MIN**	**MAX**	**X**	**MIN**	**MAX**	**X**	**δ**	**MIN-MAX**	**X**	**δ**
M1	25.0–49.3	32.0	36.0	33.5	0.19	37.2	49.3	43.9	30.0	39.0	34.5	0.25	25.0	25.0	-
M2	18.0–28.9	18.0	23.0	20.5	0.21	20.9	28.9	25.6	19.0	28.0	21.9	0.27	18.0	18.0	-
LP	7.0–20.4	12.0	14.0	13.0	0.05	14.9	20.4	18.4	8.0	17.0	1.25	0.22	7.0	7.0	-
M3	0.09–0.31	0.09	0.31	0.15	0.02	0.10	0.20	0.15	0.12	0.18	0.14	0.02	0.12	0.12	-
M4	0.39–0.74	0.39	0.60	0.50	0.08	0.50	0.70	0.63	0.49	0.74	0.61	0.07	0.56	0.56	-
M5	0.15–0.49	0.15	0.24	0.2	0.04				0.25	0.49	0.37	0.06	0.35	0.35	-
M6	0.33–0.64	-	-	-	-				0.33	0.64	0.49	0.09	0.50	0.50	-
M7	1.10–1.62	-	-	-	-				1.10	1.57	1.33	0.14	1.62	1.62	-
M8	1.61–3.81	1.61	2.22	1.90	0.23	2.88	3.81	3.19	2.23	3.23	2.65	0.23	2.86	2.86	-
M9	0.22–1.23	0.22	0.22	0.22	-				0.53	1.23	0.93	0.20	0.63	0.63	-
M10	0.01–0.08	0.01	0.06	0.02	0.02				0.04	0.08	0.06	0.01	0.07	0.07	-
M11	0.04–0.09	0.05	0.09	0.07	0.02				0.04	0.08	0.06	0.01	0.05	0.05	-
M12	0.06–0.11	0.09	0.11	0.09	0.01				0.06	0.09	0.07	0.01	0.07	0.07	-
M13	1.79–5.19	1.79	1.95	1.88	0.07				2.90	5.19	4.06	0.58	3.31	3.31	-
M14	1.07–2.34	1.07	1.07	-	-				1.32	2.34	1.97	0.29	1.74	1.74	-
M15	1.10–2.75	1.10	1.10	-	-				1.55	2.75	2.20	0.36	1.35	1.35	-

*M1, total body length of adult worm; M2, length of the esophageal region of the body; LP, length of the posterior region of the body; M3, width of the esophageal region of the body; M4, maximum width of the posterior region of the body (thickness); M5, body width in the place of the junction of the esophagus and the intestine; M6, Distance from the head end to beginning of bacillary stripes; M7, length of bacillary stripes; M8, length of spicule; M9, maximum length of spicule sheath; M10, width of the proximal end of the spicule; M11, width of the spicule sheath at the tail end of the body; M12, maximum width of the spicule sheath; M13, distance between the posterior part of the testis and tail end of body; M14, length of ejaculatory duct; M15, length of distal cloacal tube. 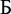, arithmetic mean. σ, standard deviation*.

The genital apparatus of the male is a long tube whose sections differ from each other in structure bearing different functions (Figure 5A). The first section of the genital apparatus is the testis, which is very long and strongly convoluted, beginning in the posterior part of the male body, directed anteriorly, and lying along the long axis of the body terminating at a short distance from the transition of the esophagus into the intestine (Figure 5A). The testis ends near the union of the ejaculator conduct and intestine. The testis is followed by the vas deferens which at first runs somewhat anteriorly along the intestine, and then, at the level of the esophageal end and somewhat short of it, describes a convolution, turns backward, and terminates in a small constriction, connecting it with the following section of the genital apparatus, the seminal vesicle. The seminal vesicle runs parallel to the intestine but does not describe sharp convolutions and via a narrow tube with thick muscular walls joins the ejaculatory duct, which is 1.07–2.34 mm long (Figure 5A). The ejaculatory duct joins the intestine to form the cloaca, which opens at the posterior end of the male body. The cloaca with anus subterminal and one pair of paracloacal papilla not ornamented (Figure 4F) was observed when the spicule sheath was invaginated. No cluster of papillae was observed. The proximal cloacal tube is wide and continued with the distal cloacal tube (1.10–2.75 mm) that contains the spicule which projects into the anterior portion of the body in a spicule tube (Figure 5A). There is only one spicule, which is elongated with a pointed tip. This spicule presents two chitinized extreme zones and a light central part and is 1.61–3.81 mm long (Figures 4E, 5A). The spicule is surrounded by a peculiar spicule sheath (0.22–1.23 mm long), which may protrude externally together with the spicule (Figure 4G). The maximum width of the spicule sheath is 0.06–0.11 mm and is covered throughout its length by densely located chitinous spines from the proximal to distal portion and is cylindrical without a distal bulb (Figures 4G, 5C and Table 3).

**Figure 6:**
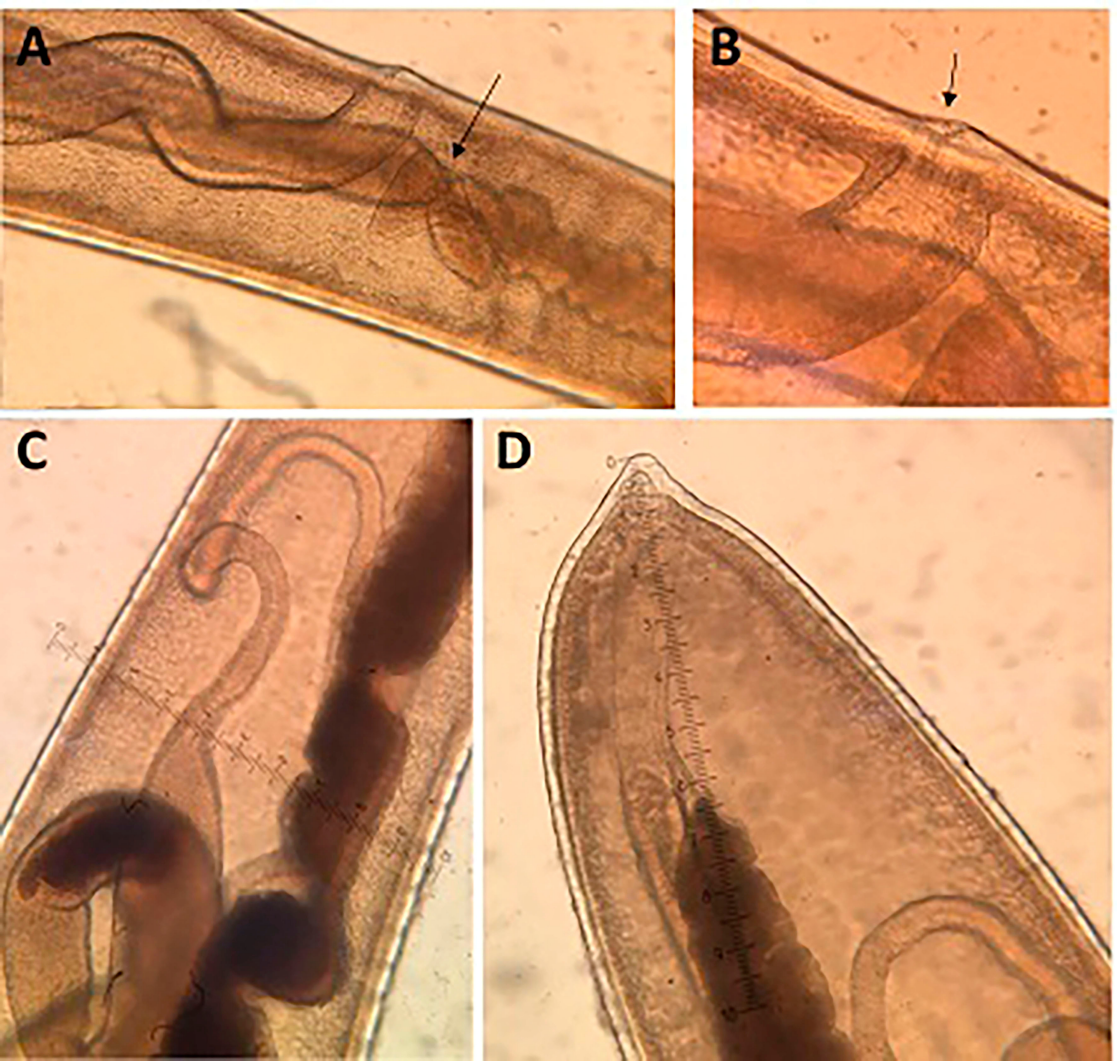
Morphology of females of *T. trichiura*. **(A)** Esophagus–intestinal junction level (arrowed), vulva and vagina. **(B)** Vulva (arrowed). **(C)** Oviduct. **(D)** Posterior end.

*Female:* The body is 20–48.6 mm long. The ratio between anterior and posterior body length is 2.1–2.2:1. The thin anterior part is 1: 0.65–0.67 of the entire length of the body. The length of the esophagus is 13–33 mm. The width of the body around the esophageal end is 0.13–0.48 mm, and the maximum width (in the posterior part of the body) is 0.38–0.90 mm (Table 4). The uterus is unpaired. The vulva is located at esophagus–intestine junction level (Figures 5B, 6A,B). This part of body thereafter changes into a transversely striated body cuticle. The vulva is non-protrusive and has no ornamentation (Figures 6A,B). The vagina has strong walls and, when everted, shows small papillae (Figure 6B). This vagina is long and presents one zone straight near the vulva while presenting circumvolutions nearly the uterus (Figures 5B, 6A). The ovary is long and continues with the oviduct in the back of the body (Figure 6C). The anus lies, subterminal, at the tip of the tail (Figures 5D, 6D). The eggs are barrel-shaped with clear, mucoid-appeared polar plugs, in addition to a vitelline membrane, and have a triple shell, the outermost layer of which is brown (Figure 5E).

**Table 4:** Biometrical data of females of *T. trichiura*.

	***T. trichiura* (Present study, updated)**	***T. trichiura*** **from** ***P. troglodytes*** **[Cutillas et al**. **(****7****)]**				***T. trichiura*** **from** ***H. sapiens*** **[Nissen et al**. **(****14****)]**			***T. trichiura*** **from** ***M. sylvanus*** **[Garc**í**a-Sánchez et al**. **(****21****)]**				***T. trichiura*** **from** ***P. papio*** **(Present study)**			
	**MIN-MAX**	**MIN**	**MAX**	**X**	**δ**	**MIN**	**MAX**	**X**	**MIN**	**MAX**	**X**	**δ**	**MIN**	**MAX**	**X**	**δ**
F1	20–48.6	20.0	42.0	33.4	0.78	29.1	48.6	38.4	30.0	38.0	34.1	0.25	24.0	28.0	26	0.28
F2	13–33	13.0	33.0	25.3	0.68	16.2	32.1	25.6	18.0	26.0	21.9	0.23	17.0	20.0	18.5	0.21
LP	6–15.60	6.0	10.0	8.1	0.14	10.5	15.60	13.7	09.0	14.0	12.1	0.16	7	8	7.5	0.07
F3	0.09–0.20	0.09	0.19	0.14	0.04	0.11	0.20	0.17	0.13	0.18	0.15	0.01	0.14	0.15	0.14	0.00
F4	0.38–0.90	0.38	0.64	0.45	0.08	0.52	0.90	0.73	0.64	0.81	0.72	0.05	0.60	0.63	0.62	0.02
F5	0.13–0.48	0.13	0.23	0.17	0.03				0.36	0.48	0.42	0.03	0.31	0.41	0.36	0.07
F6	0.38–0.76	0.48	0.64	0.56	0.11				0.42	0.76	0.50	0.09	0.38	0.42	0.40	0.03
F7	0.36–1.71	0.36	0.63	0.50	0.19				0.90	1.71	1.44	0.21	1.08	1.35	1.22	0.19
F8	0.68–1.99	0.68	1.29	0.96	0.20				0.73	1.99	1.12	0.35	0.87	0.91	0.89	0.03
F9	0.02–0.09	0.03	0.05	0.04	0.01				0.02	0.09	0.05	0.02	0.05	0.05	0.05	0.00
F10	0.33–0.11	0.11	0.24	0.15	0.05				0.15	0.33	0.25	0.05	0.18	0.19	0.18	0.01
F11	0.13–0.84	0.13	0.24	0.21	0.04				0.40	0.84	0.61	0.14	0.35	0.37	0.36	0.01
F12	0.19–0.62	0.32	0.53	0.44	0.11				0.19	0.48	0.30	0.09	0.56	0.62	0.59	0.04
F13	0.05–0.81	0.66	0.67	0.67	0.01				0.05	0.14	0.11	0.04	0.73	0.81	0.77	0.06

*F1, total body length of adult worm; F2, length of the esophageal region of the body; LP, length of the posterior region of body; F3, width of the esophageal region of the body; F4, maximum width of the posterior region of the body (thickness); F5, body width in the place of the junction of the esophagus and the intestine; F6, distance from the head end to beginning of bacillary stripes; F7, length of bacillary stripes; F8, length of vagina; F9, diameter of vulva turned over the surface of the body; F10, distance of vulva from the place of junction of the esophagus and the intestine; F11, distance of the posterior loop of the uterus from the tail end of the body; F12, distance of the tail end of the body and posterior fold of the seminal receptacle; F13, length of the muscular zone of the esophagus. 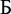, arithmetic mean. σ, standard deviation*.

## Discussion

In the present paper, we address an updated morphological and biometric description of *T. trichiura*, based on the results provided in the present study as well as on previous studies by different authors who characterized this species combining the analysis of morphological, biometric, and molecular characteristics. Thus, for the emendation of the description of *T. trichiura*, we have considered the populations of *P. troglodytes* (7), *H. sapiens* (14), *M. sylvanus* (17, 21), and *P. papio* (present study). For many years, different authors have based on the description of *T. trichiura* isolated from humans and NHP, on morphological and biometric characteristics exclusively (6, 25, 39–43), which was not molecularly confirmed to have corresponded to *T. trichiura*.

Thus, we found that females of *T. trichiura* are characterized by a non-protrusive vulva without ornamentation. However, some authors have described the vulva with a surface covered with spines like those of the male's spicule sheath (6, 25). Likewise, based on the morphological characteristics of males, *T. trichiura* is characterized by the presence of a lanceolate spicule that tapers at the end (41). However, Tenora et al. (42) observed a spicule with a cylindrical end in isolated samples of *H. sapiens*. Furthermore, males of *T. trichiura* isolated from *P. papio* and *M. sylvanus* presented a spicule with two chitinized extreme zones and a lighter central part (17). This feature was not observed in males of *T. trichiura* described by several authors who exclusively carried out morphological and biometric studies (6, 7, 25, 39–42).

On the other hand, the spicule sheath is cylindrical without a distal bulb with triangular spines; however, other authors described these spines with different shapes and sizes, some of them with blunt points in *T. trichiura* from humans and NHP (25, 42). Besides, we found that the males of *T. trichiura* are characterized by the presence of a pair of paracloacal papillae which are observed when the sheath is invaginated (17); however, a proximal group of small papillae is not observed, as described by Ooi et al. (6) in a morpho-biometric study carried out on samples of monkeys, baboons, and humans. These papillae cluster was described in *T. colobae* by Cutillas et al. (19) who characterized a new species of *Trichuris* present in *C. g. kikuyuensis* based on morphological, biometric, and molecular data. This fact could suggest that the descriptions carried out by Ooi et al. (6) of *Trichuris* populations from humans and NHP based exclusively on morphological and biometric characters could correspond to different species closed to *T. trichiura*.

Biometrically, there is a concordance between the measures of *T. trichiura* obtained by different authors and those provided in the present paper, since all the values overlapped within the range of defined measures. However, regarding males, Dinnik et al. (40) reported that the maximum total body length and the maximum length of the anterior part [total body length of adult worm (M1) and length of esophageal region of body (M2)] were slightly larger (52 and 34 mm, respectively). Furthermore, the maximum width of the posterior region of body (thickness) (M4), length of spicule (M8), and length of ejaculatory duct (M14) has also been reported as slightly higher by Skrjabin et al. (25) (M4 = 0.76 mm, M8 = 3.9 mm, and M14 = 3.9 mm). Regarding females, the maximum total body length of adult worm (M1) showed slightly higher values in the studies of Dinnik et al. (40), Skrjabin et al. (25), and Vigot-Fréres (39) (50, 52.7, and 50 mm, respectively). In addition, the maximum length of the esophageal region of body (M2) and the maximum body width in the place of junction of esophagus and the intestine (F5) were slightly higher (35.6 and 0.50 mm, respectively) in the data provided by Skrjabin et al. (25).

In addition, there are differences in the population of *Trichuris* sp. from chimpanzees showing a shorter size of the males and females collected from chimpanzees with respect to *T. trichiura* from *H. sapiens, M. sylvanus*, and *P. papio*. García-Sánchez et al. (21) who reported the differentiation of *Trichuris* species using a morphometric approach cited these results previously. The occurrence of different biometrical measurements in the same species may be explained by the phenotypic plasticity of these organisms themselves (8–11).

Morphological and biometric data for *T. trichiura* provided in the present study allow the differentiation of this species with respect to other species of *Trichuris* parasitizing NHP such as *T. colobae* (19) and *T. ursinus* (20). The typical *T. trichiura* spicule has a clear central part, which is not present in that of *T. colobae* and *T. ursinus*. Furthermore, the typical papilla group is only present in *T. colobae* (19). Females of *T. trichiura* and *T. ursinus* appear to have a non-protrusive vulva (20); nevertheless, females of *T. colobae* present a vulva like a crater with papillae (19). The vagina is very long and straight in *T. ursinus* (20) but appear with circumvolutions in *T. trichiura*.

With respect to the biometric characteristics, most of the measurements do not allow the specific differentiation between *T. trichiura* and other *Trichuris* spp. of NHP since these values overlapped for most of the measurements. However, males of *T. trichiura* have a smaller range of values in terms of distance from the end of the head to beginning of bacillary stripes and length of bacillary stripes (M6 and M7) compared to *T. colobae*, and values of length of minor bacillary stripes regarding *T. ursinus*. Regarding the specific differentiation of females based on biometric measurements, *T. trichiura* presents a lower range of values of length of bacillary stripes and distance of the tail end of the body and posterior fold of the seminal receptacle (F7 and F12) respect to *T. colobae*. In addition, the maximum and minimum values of distance of the vulva from the place of junction of the esophagus and the intestine were below than those observed in *T. ursinus* (19, 20).

From a molecular point of view, molecular markers which have been used by different authors to resolve species-level questions in *Trichuris* include the ITS1 and ITS2 nuclear regions (7, 44–53), 18S nuclear rRNA gene (38, 53, 54), mtDNA 16S rRNA gene (*rrn*L) (17, 50, 55), and protein-coding mitochondrial genes, including the 13 common genes obtained from the mitochondrial complete genome (12, 16, 56), *cox*1 mtDNA partial gene (17, 20, 38, 53, 55, 57), and *co*b mtDNA partial gene (17, 20, 55, 57, 58).

Different ITS rDNA types have been cited by different authors in some species of *Trichuris* (45–47). These sequence differences among ITS repeats in the rDNA array appeared to be a consequence of (intrachromosomal) mutational exchange during DNA replication (59). Thus, different reports suggest that different sequence types are most likely to be a result of base changes at certain positions in the sequence of a proportion of rDNA repeats because of mutational exchange during DNA replication, the extent of which appears to differ depending on the taxonomic group (60–63).

The results observed in ITS sequences revealed a percentage of similarity between 2a, 2b, 2c, and 2d subclades higher than those previously observed by other authors for species of the genus *Trichuris* (50, 57). Genetic analysis revealed that ITS regions were saturated and showed poor nucleotide diversity for clade 2 (*T. trichiura*). Thus, ITS sequences were not useful to infer the phylogenetic relationships between the different populations of *T. trichiura* (clade 2). On the other hand, some sequences from *T. trichiura* from Uganda and Cameroon (4) appeared within clade 1, suggesting that this population could be included on *T. ursinus* due to the high interpopulation similarity observed between both populations (Supplementary Table 4 and Figure 1). Therefore, the different genetic lineages within *T. trichiura* were delimited exclusively based on analysis of the mitochondrial genes in agreement with Chan et al. (64), who evaluated the utility of mitochondrial and ribosomal genes for molecular systematics of parasitic nematodes. ITS regions accumulated substitutions substantially more slowly than mtDNA and showed nucleotide saturation.

Studies of interpopulation similarity between the different 2a, 2b, 2c, and 2d subclades, based on the mitochondrial markers (*cox*1 and *co*b), revealed a range of similarity lower than those observed by ITS sequences, and similar to those observed among other clearly defined species such as for example *T. suis* and *T. colobae, T. colobae*, and *T. ursinus* [cited by present authors, Supplementary Tables 5, 6 and previously by Callejón et al. (20) and Rivero et al. (17)]. However, this similarity range showed higher values between *rrn*L sequences presenting a strong resolution of the different *T. trichiura* lineages (Supplementary Table 7). These results agree with those of Chan et al. (64), who evaluated the utility of mitochondrial ribosomal genes for molecular systematics of parasitic nematodes. These authors cited that 18S and 28S rRNA genes as well as 12S (*rrn*S) and 16S (*rrn*L) rRNA and *cox*1 genes showed a higher resolution for phylogenetic studies indicating that these five genes have potentially to be used as markers. Furthermore, they demonstrated that mitochondrial 12S and 16S genes present a resolution power at both lower and higher taxonomic levels for species and clade discrimination (64). For this reason and considering the similarity values observed in the different markers, we suggest that the populations of *T. trichiura* corresponding to the 2a, 2b, 2c, and 2d subclades correspond to different genetic lineages.

On the other hand, within clade 2 “*T. trichiura* lineage,” results based on *rrn*L revealed an interpopulation similarity of 83.80–86.31% between *T. trichiura* populations and *Trichuris* sp. from *T. francoisi*. Hence, we suggest that this species has a close relationship with *T. trichiura*. Our results agree with Liu et al. (12) who considered that *Trichuris* populations from *T. francoisi* are a new species of *Trichuris*.

The phylogeny inferred from mitochondrial datasets revealed the same topology of those based on rDNA with respect to the two main clades (clade 1 and clade 2). We were able to identify several genetically distinct subgroups (subclades) of whipworms, which were present in the sampled primates. The subclades 2b and 2c showed a broad host range and were not restricted to NHP species. However, the subclades 2a and 2d showed a higher host specificity corresponding with the *T. trichiura* population from *M. sylvanus* and *H. sapiens* (subclade 2a) and *M. fuscata* (subclade 2d) exclusively. In agreement to this study, similar results were observed on *Trichuris* sp. from *M. fuscata* (44, 55). This population also showed two potentially distinct entities of *Trichuris* present in two different subclades: subclade 2d [analogous to subclade MF reported by Cavallero et al. (44, 55)] and subclade 2c. These authors suggested the possibility of two different sources of infection for Japanese macaques corresponding with two *Trichuris* taxa, one potentially able to also infect humans.

In our study, all phylogenetic trees (partitioned and concatenated) reported the existence of two main clades, which has been previously reported (3, 7, 12, 17, 20, 33, 44, 55, 58). A clear differentiation between *T. suis* (clade 1) and *T. trichiura* (clade 2) can be confirmed according to our results. Within clade 2 “*T. trichiura* lineage,” *T. trichiura* can be also divided into 4 subclades, suggesting a complex of different genetic lineages. The analysis of the intraspecific similarity between the populations of *T. trichiura* from *P. papio, C. aethiops*, and *E. patas* from Spain revealed their highest value when compared with the populations of *T. trichiura* belonging to subclade 2c [previously described by Rivero et al. (17)] in all analyzed markers.

In addition to *T. trichiura*, a complex of *Trichuris* species showed infecting primates (*Trichuris* sp. from *T. francoisi* within clade 2 “*T. trichiura* lineage” and from *C. aethiops* from Italy and *C. sabaeus* from Czech Republic within clade 1 “*T. suis* lineage”). Different authors (17, 65) have previously reported the presence of several groups of *T. trichiura*. These results emphasize that the taxonomy and genetic variations of *Trichuris* are more complicated than previously acknowledged (65). Ravasi et al. (3) identified two distinct *Trichuris* genotypes that infect both humans and NHP. These authors identified “heterozygotes” confirming the identification of two distinct *Trichuris* genotypes in primates. On the other hand, Nissen et al. (14) identified “heterozygote” worms isolated from humans suggesting that *T. trichiura* might consist of several subspecies, some being found mainly in NHP. Ghai et al. (66) suggested that the *Trichuris* taxon should be considered a multi-host pathogen that is capable of infecting wild primates and humans. Finally, Betson et al. (5) reported that *Trichuris* infecting primates represents a complex of cryptic species with some species being able to infect both humans and NHP.

On the other hand, the existence of populations of *Trichuris* sp. associated with clade 1 “*T. suis* lineage” (based on all partitioned and concatenated markers) could indicate the possible origin of the change of host from NHP to pigs and, therefore, the origin of a new species, *T. suis*. This fact has been suggested by Hawash et al. (16) who found evidence of an African origin of *T. trichiura* that was later transmitted with human ancestors to Asia and then to South America. These authors suggested the possibility of a change of host to pigs in Asia where *T. suis* appears to have been transmitted globally by a combination of natural host dispersion and anthropogenic factors.

The present work examines the taxonomy, genetics, and phylogeny of *T. trichiura* parasitizing human and NHP and its relationship to *Trichuris* spp. from other NHP host species. A similar analysis was carried out on whipworms from humans (5). These authors suggested the existence of zoonotic transmission, especially regarding *T. trichiura* infections in NHP and, possibly, also for *T. suis* from pigs and *Trichuris vulpis* from dogs. In consequence, *Trichuris* may represent different species with the potential differences in endemicity, which may have important implications for implementing effective control strategies (5).

Several studies have reported that NHP represent an important reservoir for several known zoonotic infectious diseases (67–69). In this context, *Trichuris* infections have been found in a range of NHP species living in natural habitats including colobus monkeys, macaques, baboons, and chimpanzees (70–78). Based on molecular studies described above, some *Trichuris* species seem to be specific to a particular NHP, while others likely have the potential to circulate between humans and NHP, as they are genetically identical. This is particularly important when humans and NHP live in close proximity, as it is becoming increasingly common with human encroachment on pristine habitats and NHP accessing gardens and farms in search of food, and it has significant implications for human health and wildlife conservation (5).

The taxonomic, genetic, and phylogenetic results obtained in the present study confirm that *T. trichiura* exists as a complex (four subclades) with different host affinities and cross-infection capabilities corresponding with four different genetic lineages. Specifically, two of the four subclades show little host specificity and can develop trichuriasis in a wider variety of NHP species (*C. sabaeus, C. aethiops, M. fuscata, M. sylvanus, E. patas, P. papio, P. hamadryas, Papio* sp., and *P. anubis*) shared with humans. For this reason, we suggest the existence of a possible reservoir in the previously mentioned NHP species for human trichuriasis, which constitutes a serious public health risk (17). However, previous studies showed that the majority of the population of *M. fuscata* was included in a specific subclade [2d, subclade MF cited by Cavallero et al. (44, 55)] including only specimens from macaques.

Therefore, considering the following arguments for the taxonomic and phylogenetic study of populations of *T. trichiura*: (i) ITS regions were saturated and accumulated substitutions substantially slowly than mtDNA; therefore, they are not good genetic markers to delimit different genetic lineages. This is in agreement with previous studies where intraspecific variation was not observed when using nuclear DNA (64) suggesting that is not a useful genetic marker for intraspecies discrimination. (ii) The mitochondrial genes *cox*1, *co*b, and *rrn*L were not saturated, indicating that these three genes could have potential to be used as markers. Nevertheless, although *cox*1 presents the advantage for the extensive availability of database sequences, allowing for thorough comparisons of unknown sequences, *cox*1 sequences showed a high intraspecific variability which hinders resolution between closely related species. (iii) Nucleotide diversity showed that the *rrn*L gene present a lower intraspecific variability than *cox*1 and *co*b genes. However, *rrn*L interspecific genetic distance values allowed the phylogenetic resolution of the different *T. trichiura* subclades. Whence, *rrn*L was used successfully for inter-lineage discrimination of closely related populations within the *T. trichiura* lineage.

We suggest the utility of *rrn*L rRNA gene as a useful genetic marker for *Trichuris* species discrimination. Different authors who evaluated the utility of different mitochondrial genes for phylogenetic analyses (64, 79–81) have cited similar results. Future studies should focus on developing sequence databases for rRNA genes, which can serve as alternative genetic.

In conclusion, the present work provides an extensive study of biometric, morphological and molecular data for the unification of criteria that allows an update description of *T. trichiura* as well as a complex taxonomic, genetic, and phylogenetic study of the cited species applying multiple genetic markers to whipworm populations collected from humans and NHP from sympatric areas and worldwide locations. This study provides useful results for future studies aimed at the identification of new subspecies and hybridization events between existing species and enables a much clearer and more detailed understanding of dispersal patterns. This will reveal parasite transmission routes between these primates and will allow the implementation of appropriate control and prevention measures.

## Data Availability Statement

The datasets presented in this study can be found in online repositories. The names of the repository/repositories and accession number(s) can be found in the article/Supplementary Material.

## Author Contributions

JR, RC, and CC contribute conception, design of the study, and wrote the manuscript. All the authors contributed to the manuscript revision and read and approved the submitted version.

## Conflict of Interest

The authors declare that the research was conducted in the absence of any commercial or financial relationships that could be construed as a potential conflict of interest. The handling Editor declared a past co-authorship with several of the authors CC and RC.
