# Supplementary material for: Trichuris trichiura (Linnaeus, 1771) From Human and Non-human Primates: Morphology, Biometry, Host Specificity, Molecular Characterization, and Phylogeny
Source: Front Vet Sci. 2021 Feb 9;7:626120. doi: 10.3389/fvets.2020.626120 (PMC7934208; doi:10.3389/fvets.2020.626120)
Supplement: Supplementary file 1 [file Table_1.DOCX]

|  | ITS | *cox*1 | *co*b | *rrn*L |
| --- | --- | --- | --- | --- |
| PCR Mix | | | | |
| Forward Primer (10 µM) | 5 µl | 5 µl | 5 µl | 5 µl |
| Reverse Primer (10 µM) | 5 µl | 5 µl | 5 µl | 5 µl |
| GoTaq G2 Green Master Mix | 25 µl | 25 µl | 25 µl | 25 µl |
| Template DNA | 5 µl | 5 µl | 5 µl | 5 µl |
| Nuclease free water to | 50 µl | 50 µl | 50 µl | 50 µl |
| PCR Primers | | | | |
| Forward Primer | NC5 (Gasser et al., 1996) | HC02198F (Folmer et al., 1994) | D769 (Callejón et al., 2015) | TTrrnLF (Liu et al., 2012) |
| Reverse Primer | NC2 (Gasser et al., 1996) | CORA (Nagano et al., 1999) | D770 (Callejón et al., 2015) | TTrrnLR (Liu et al., 2012) |
| PCR Conditions | | | | |
| Initial Denaturing | 94° C/10 min | 94° C/5 min | 94° C/5 min | 95° C/15 min |
| Number of cycles | 35 | 40 | 36 | 35 |
| Denaturing | 94° C/1 min | 94° C/1min | 94° C/30 s | 95° C/30 s |
| Annealing | 55° C/1 min | 48° C/1min | 50° C/30 s | 55° C/30 s |
| Primer extension | 72° C/1 min | 72° C/1min | 72° C/30 s | 72° C/1 min |
| Final extension | 72° C/10 min | 72° C/7min | 72° C/5 min | 72° C/10 min |

**Table S1.** PCR mix, primers and conditions used for each molecular marker sequenced in the present study.

**References**

Callejón R, Gutiérrez-Avilés L, Halajian A, Zurita A, de Rojas M, Cutillas C. Taxonomy and phylogeny of *Trichuris globulosa* Von Linstow, 1901 from camels. A review of *Trichuris* species parasitizing herbivorous. Infect Genet Evol. (2015) 34:61-74. doi: 10.1016/j.meegid.2015.06.011.

Folmer O, Black M, Hoeh W, Lutz, R, Vrijenhoek, R. DNA primers for amplification of mitochondrial *cytochrome c oxidase* subunit I from diverse metazoan invertebrates. Mol. Mar. Biol. Biotechnol. (1994) 3: 294-299. PMID: 7881515.

Gasser RB, Stewart LE, Speare R. Genetic markers in ribosomal DNA for hookworm identification. Acta Trop. (1996) 62:15-21. doi: 10.1016/s0001-706x(96)00015-0.

Liu GH, Gasser RB, Su A, Nejsum P, Peng L, Lin RQ, et al. Clear genetic distinctiveness between human-and pig-derived *Trichuris* based on analysis of mitochondrial datasets. PLoS Negl Trop Dis. (2012) 6:e1539. doi: 10.1371/journal.pone.0066249.

Nagano I, Wu Z, Matsuo A, Pozio E, Takahashi Y. Identification of *Trichinella* isolates b polymerase chain reaction-restriction fragment length polymorphism of the mitochondrial cytochrome c-oxidase subunit I gene. Int. J. Parasitol. (1999) 29: 1113-1120. doi.org/10.1016/S0020-7519(99)00060-0
