# Supplementary material for: Trichuris trichiura (Linnaeus, 1771) From Human and Non-human Primates: Morphology, Biometry, Host Specificity, Molecular Characterization, and Phylogeny
Source: Front Vet Sci. 2021 Feb 9;7:626120. doi: 10.3389/fvets.2020.626120 (PMC7934208; doi:10.3389/fvets.2020.626120)
Supplement: Supplementary file 2 [file Table_2.docx]

**Table S2.** Sequences of *Trichuris* spp. and outgroups species obtained from GenBank and used for phylogenetic analysis. * Samples analyzed in the present study. The new ITS2 sequences from this study underlined are the haplotypes used to make the ribosomal concatenated phylogenetic tree.

| Species | Host species/ Geographical origin | Marker | Accession number |
| --- | --- | --- | --- |
| *Trichuris colobae* | *Colobus guereza kikuyensis*/Spain | *cox*1 | HE653116 |
|  |  |  | HE653118 |
|  |  |  | JF690968 |
|  | *Colobus guereza kikuyensis*/Italy |  | MK762948 |
| *Trichuris* sp. | *Chlorocebus aethiops*/Italy |  | MK762929 |
|  |  |  | MK762931 |
|  | *Chlorocebus sabaeus*/Czech Republic |  | MK762923 |
|  |  |  | MK762924 |
|  |  |  | MK762925 |
|  |  |  | MK762927 |
|  | *Colobus guereza kikuyensis*/Czech Republic |  | JF690968 |
|  | *Homo sapiens*/Czech Republic |  | JF690962 |
|  | *Macaca fuscata*/Europe |  | MK762905 |
|  |  |  | MK762906 |
|  |  |  | MK762908 |
|  |  |  | MK762909 |
|  |  |  | MK762915 |
|  |  |  | MK762919 |
|  |  |  | MK762920 |
|  |  |  | MK762921 |
|  | *Papio anubis*/USA |  | KT449825 |
|  | *Papio hamadryas*/Czech Republic |  | JF690963 |
|  | *Papio hamadryas*/Denmark |  | KT449824 |
|  | *Papio hamadryas*/Europe |  | MK762943 |
|  |  |  | MK762945 |
| *Trichuris trichiura* | *Chlorocebus aethiops*/Spain |  | LR898048* |
|  | *Erythrocebus patas*/Spain |  | LR898049* |
|  | *Homo sapiens*/China |  | GU385218 |
|  |  |  | NC_017750 |
|  | *Homo sapiens*/Uganda |  | KT449826 |
|  | *Homo sapiens* /Japan |  | AP017704 |
|  | *Macaca sylvanus*/Spain |  | LR130781 |
|  |  |  | LR130782 |
|  |  |  | LR130783 |
|  |  |  | LR130784 |
|  | *Papio papio*/Spain |  | LR898050* |
|  | *Papio* sp./Spain |  | HG003692 |
| *Trichuris suis* | *Sus scrofa*/China |  | NC_017747 |
|  | *Sus scrofa domestica*/Spain |  | HE653124 |
|  | *Sus scrofa domestica*/Denmark |  | KT449822 |
|  | *Sus scrofa domestica*/Uganda |  | KT449823 |
|  | *Sus scrofa domestica*/China |  | HQ204208 |
|  |  |  | HQ204210 |
|  |  |  | HQ204209 |
|  |  |  | GU070737 |
|  | *Sus scrofa scrofa*/Spain |  | HE653127 |
| *Trichuris ursinus* | *Papio ursinus*/South Africa |  | LT627353 |
| *Trichuris colobae* | *Colobus guereza kikuyensis*/Spain | *co*b | LM994704 |
|  |  |  | MK914578 |
| *Trichuris* sp. | *Chlorocebus aethiops*/Italy |  | MK914564 |
|  |  |  | MK914570 |
|  |  |  | MK914571 |
|  | *Chlorocebus sabaeus*/Czech Republic |  | MK914562 |
|  |  |  | MK914563 |
|  | *Macaca fuscata/*Europe |  | MK914550 |
|  |  |  | MK914551 |
|  |  |  | MK914554 |
|  |  |  | MK914555 |
|  |  |  | MK914556 |
|  |  |  | MK914557 |
|  |  |  | MK914560 |
|  | *Papio anubis*/USA |  | KT449825 |
|  | *Papio hamadryas*/Czech Republic |  | MK914573 |
|  |  |  | MK914574 |
|  |  |  | MK914575 |
|  |  |  | MK914576 |
|  |  |  | MK914577 |
|  | *Papio hamadryas*/Denmark |  | KT449824 |
|  | *Papio* sp./Spain |  | LM994703 |
| *Trichuris trichiura* | *Chlorocebus aethiops*/Spain |  | LR898051* |
|  | *Erythrocebus patas*/Spain |  | LR898052* |
|  | *Homo sapiens*/China |  | GU385218 |
|  |  |  | NC_017750 |
|  | *Homo sapiens*/Uganda |  | KT449826 |
|  | *Macaca sylvanus*/Spain |  | LR132031 |
|  |  |  | LR132032 |
|  |  |  | LR132033 |
|  |  |  | LR132034 |
|  |  |  | LR757999* |
|  | *Papio papio*/Spain |  | LR898053* |
|  |  |  | LR898054* |
|  |  |  | LR898055* |
|  |  |  | LR898056* |
| *Trichuris suis* | *Sus scrofa*/China |  | NC_017747 |
|  | *Sus scrofa domestica*/China |  | GU070737 |
|  | *Sus scrofa domestica*/Denmark |  | KT449822 |
|  | *Sus scrofa domestica*/Uganda |  | KT449823 |
|  | *Sus scrofa scrofa*/Spain |  | LM994696 |
| *Trichuris ursinus* | *Papio ursinus*/South Africa |  | LT627357 |
|  |  |  | LT627358 |
|  |  |  | LT627359 |
|  |  |  | LT627360 |
| *Trichuris colobae* | *Colobus guereza kikuyensis*/Spain | *rrn*L | MN088583 |
|  |  |  | MN088584 |
|  |  |  | MN088585 |
|  | *Colobus guereza kikuyensis*/Spain |  | LR898058* |
|  |  |  | LR898059* |
|  |  |  | LR898060* |
|  |  |  | LR898061* |
|  |  |  | LR898062* |
| *Trichuris* sp. | *Chlorocebus aethiops*/Italy |  | MN088565 |
|  | *Chlorocebus sabaeus*/Czech Republic |  | MN088559 |
|  |  |  | MN088560 |
|  |  |  | MN088561 |
|  | *Chlorocebus sabaeus*/Saint Kitts and Nevis |  | KU524595 |
|  |  |  | KU524599 |
|  |  |  | KU524600 |
|  |  |  | KU524601 |
|  |  |  | KU524602 |
|  | *Macaca fuscata*/Italy |  | MN088542 |
|  |  |  | MN088543 |
|  |  |  | MN088544 |
|  |  |  | MN088546 |
|  |  |  | MN088551 |
|  |  |  | MN088553 |
|  |  |  | MN08855 |
|  |  |  | MN088557 |
|  | *Papio anubis*/USA |  | KT449825 |
|  | *Papio hamadryas*/Czech Republic |  | MN088578 |
|  |  |  | MN088580 |
|  | *Papio hamadryas*/Denmark |  | KT449824 |
|  | *Papio* sp./Denmark |  | KU524558 |
|  |  |  | KU524559 |
|  |  |  | KU524564 |
|  |  |  | KU524573 |
|  |  |  | KU524574 |
|  |  |  | KU524575 |
|  |  |  | KU524576 |
|  |  |  | KU524581 |
|  |  |  | KU524584 |
|  | *Trachypithecus francoisi*/China |  | KC481232 |
|  |  |  | KC481233 |
|  |  |  | KC481234 |
|  |  |  | KC481235 |
|  |  |  | KC461179 |
| *Trichuris trichiura* | *Homo sapiens*/China |  | GU385218 |
|  |  |  | NC_017750 |
|  |  |  | AM993017 |
|  |  |  | AM993018 |
|  |  |  | AM993019 |
|  |  |  | AM993020 |
|  | *Homo sapiens*/Ecuador |  | KP781898 |
|  |  |  | KP781899 |
|  |  |  | KP781900 |
|  |  |  | KP781901 |
|  |  |  | KP781906 |
|  | *Homo sapiens*/Uganda |  | KT449826 |
|  |  |  | KU524541 |
|  |  |  | KU524542 |
|  |  |  | KU524544 |
|  |  |  | KU524545 |
|  |  |  | KU524548 |
|  |  |  | KU524557 |
|  | *Macaca sylvanus*/Spain |  | LR898063* |
|  |  |  | LR898064* |
|  |  |  | LR898065* |
|  |  |  | LR898066* |
|  |  |  | LR898067* |
|  | *Papio papio*/Spain |  | LR898057* |
| *Trichuris suis* | *Sus scrofa*/China |  | NC_017747 |
|  | *Sus scrofa domestica*/China |  | GU070737 |
|  | *Sus scrofa domestica*/Denmark |  | KT449822 |
|  | *Sus scrofa domestica*/Ecuador |  | KP781894 |
|  | *Sus scrofa domestica*/Uganda |  | KT449823 |
|  |  |  | KU524537 |
|  |  |  | KU524540 |
| *Trichuris colobae* | *Colobus guereza kikuyensis*/Spain | ITS2 | FM991956 |
|  | *Nomascus gabriellae*/Spain |  | FM991955 |
| *Trichuris* sp. | *Chlorocebus aethiops*/China |  | KT344827 |
|  | *Macaca leonina*/China |  | KT344828 |
|  | *Macaca mulatta*/China |  | MH390369 |
|  |  |  | KT344829 |
|  | *Nomascus leucogenys*/China |  | KT344830 |
|  | *Papio anubis*/China |  | KT344826 |
|  | *Papio hamadryas*/China |  | KT344831 |
|  | *Papio ursinus*/South Africa |  | GQ301551 |
|  | *Rhinopithecus roxellana*/China |  | KT344825 |
|  | *Trachypithecus francoisi*/China |  | KT186231 |
|  |  |  | KT186232 |
|  |  |  | KT186233 |
|  |  |  | KT186234 |
| *Trichuris trichiura* | *Cercopithecus ascanius*/Uganda |  | KJ588135 |
|  | *Cercopithecus lhoesti*/Uganda |  | KJ588163 |
|  | *Cercopithecus mitis*/Uganda |  | KJ588156 |
|  | *Chlorocebus aethiops*/Spain |  | LR898026* |
|  |  |  | LR898027* |
|  |  |  | LR898028* |
|  |  |  | LR898029* |
|  |  |  | LR898030* |
|  | *Colobus guereza*/Uganda |  | KJ588167 |
|  | *Erythrocebus patas*/Spain |  | LR898031* |
|  | *Homo sapiens*/Cameroon |  | GQ301555 |
|  | *Homo sapiens*/China |  | AM992987 |
|  | *Homo sapiens*/Uganda |  | JN181859 |
|  | *Lophocebus albigena*/Uganda |  | KJ588147 |
|  | *Macaca sylvanus*/Spain |  | LR535741 |
|  |  |  | LR535747 |
|  |  |  | LR535749 |
|  |  |  | LR535750 |
|  |  |  | LR898025* |
|  | *Pan troglodytes*/Uganda |  | KJ588160 |
|  | *Papio anubis*/Uganda |  | KJ588152 |
|  | *Papio hamadryas*/Turkey |  | KC877992 |
|  | *Papio papio*/Spain |  | LR898032* |
|  |  |  | LR898033* |
|  |  |  | LR898034* |
|  |  |  | LR898035* |
|  |  |  | LR898036* |
|  | *Procolobus rufomitratus*/Uganda |  | KJ588162 |
| *Trichuris suis* | *Sus scrofa*/China |  | AM992999 |
|  | *Sus scrofa domestica*/Spain |  | AJ249966 |
| *Trichuris ursinus* | *Papio ursinus*/South Africa |  | GQ301554 |
| *Trichuris colobae* | *Colobus guereza kikuyensis*/Spain | ITS1 | FM991956 |
|  | *Nomascus gabriellae*/Spain |  | FM991955 |
| *Trichuris* sp. | *Chlorocebus aethiops*/China |  | KT344827 |
|  | *Macaca leonina*/China |  | KT344828 |
|  | *Macaca mulatta*/China |  | KT344829 |
|  |  |  | MH390369 |
|  | *Nomascus leucogenys*/China |  | KT344830 |
|  | *Papio anubis*/China |  | KT344826 |
|  | *Papio hamadryas*/China |  | KT344831 |
|  | *Papio ursinus*/South Africa |  | GQ301551 |
|  | *Rhinopithecus roxellana*/China |  | KT344825 |
|  | *Trachypithecus francoisi*/China |  | KT186231 |
|  |  |  | KT186232 |
|  |  |  | KT186233 |
|  |  |  | KT186234 |
| *Trichuris trichiura* | *Cercopithecus ascanius*/Uganda |  | KJ588097 |
|  | *Cercopithecus lhoesti*/Uganda |  | KJ588102 |
|  | *Cercopithecus mitis*/Uganda |  | KJ588104 |
|  | *Chlorocebus aethiops*/Spain |  | LR898012* |
|  |  |  | LR898013* |
|  | *Colobus guereza*/Uganda |  | KJ588076 |
|  | *Erythrocebus patas*/Spain |  | LR898014* |
|  | *Homo sapiens*/Cameroon |  | GQ301555 |
|  | *Homo sapiens*/China |  | AM992987 |
|  | *Homo sapiens*/Uganda |  | KJ588075 |
|  | *Lophocebus albigena*/Uganda |  | KJ588096 |
|  | *Macaca sylvanus*/Spain |  | LR898004* |
|  |  |  | LR898005* |
|  |  |  | LR898006* |
|  |  |  | LR898007* |
|  |  |  | LR898008* |
|  |  |  | LR898009* |
|  | *Pan troglodytes*/Uganda |  | KJ588094 |
|  | *Papio anubis*/Uganda |  | KJ588099 |
|  | *Papio hamadryas*/Turkey |  | KC877992 |
|  | *Papio papio*/Spain |  | LR898010* |
|  |  |  | LR898011* |
|  | *Procolobus rufomitratus*/Uganda |  | KJ588122 |
| *Trichuris suis* | *Sus scrofa*/China |  | AM992999 |
|  | *Sus scrofa domestica*/Spain |  | AJ781762 |
| *Trichuris ursinus* | *Papio ursinus*/South Africa |  | GQ301554 |
| **Outgroup species (mitochondrial)** | | | |
| *Trichinella pseudospiralis* |  |  | KM357411 |
| *Trichinella spiralis* |  |  | AF293969 |
| **Outgroup species (ribosomal)** | | | |
| *Trichinella nativa* |  |  | KP307966 |
| *Trichinella spiralis* |  |  | KC006415 |
