# Supplementary material for: Trichuris trichiura (Linnaeus, 1771) From Human and Non-human Primates: Morphology, Biometry, Host Specificity, Molecular Characterization, and Phylogeny
Source: Front Vet Sci. 2021 Feb 9;7:626120. doi: 10.3389/fvets.2020.626120 (PMC7934208; doi:10.3389/fvets.2020.626120)
Supplement: Supplementary file 3 [file Table_3.DOCX]

**Table S3**. Monophyly based on different markers (ITS1, ITS2, *cox*1, *co*b and *rrn*L) of selected group based on different combination of datasets and inference methods (MP/BPP). ML: Maximum Likelihood bootstrap; BPP: Bayesian Posterior Probability.

|  | Nuclear region ITS1 + ITS2 | *cox*1 mt gene | *co*b mt gene | *rrn*L mt gene | mt genes  (*cox*1 + *co*b) | mt and nuclear markers (*cox*1 + *co*b + ITS1 + ITS2) |
| --- | --- | --- | --- | --- | --- | --- |
| Clade 1 | 100/100 | 86/97 | -/95 | 76/100 | 100/98 | 100/100 |
| Clade 2 | 100/100 | 70/78 | 100/100 | 90/100 | 100/100 | 100/100 |
| **CLADE 2** |  |  |  |  |  |  |
| Subclade 2a | - | 86/80 | 95/99 | 100/100 | 100/100 | 100/100 |
| Subclade 2b | - | 98/97 | 91/100 | 88/99 | 100/98 | - |
| Subclade 2c | - | 98/86 | 80/90 | 90/100 | 100/100 | 100/100 |
| Subclade 2d | - | 81/- | 100/100 | 63/84 | 100/100 | - |
| *Trichuris* sp. (Subclade *T. francoisi*) | 100/100 | - | - | 99/100 | - | - |
| Subclade 2a + 2b | - | - | 100/100 | 86/100 | 100/91 | 100/87 |
| Subclade 2c + 2d | - | 98/80 | - | - | 100/84 | - |
| Subclade 2a + 2b + 2c | 100/100 | - | - | - | - |  |
| Subclade 2a + 2b + 2d | - | - | -/- | 85/100 | - | - |
| Subclade 2a + 2b + 2c + 2d | 87/100 | - | - | -/99 | - | - |
| Subclade 2a + 2b + 2c + *Trichuris* sp. (from *M. mulatta*) | 100/100 | - | - | - | - | - |
| **CLADE 1** |  |  |  |  |  |  |
| *T. colobae* | 100/100 | 86/100 | 100/100 | 97/100 | 100/100 | - |
| *T. ursinus* | - | - | 100/93 | - | - | - |
| *T. suis* | 100/100 | 98/100 | 100/100 | 99/100 | 100/100 | 100/100 |
| *Trichuris* sp. | 100/100 | 96/100 | 93/100 | 97/100 | 100/100 | - |
| *T. colobae* + *T. ursinus* | - | - | - | - |  | 100/100 |
| *T. colobae* + *T. suis* | - | - | 65/82 | - | - | - |
| *T. colobae* + *T. ursinus* + *Trichuris* sp. | 100/100 | - | - | - | 100/83 | - |
| *T. colobae* + *Trichuris* sp. + *T. suis* | - | - | - | 76/100 | - | - |
| *T. ursinus* + *Trichuris* sp. | 100/100 | -/- | -/78 | - | 100/98 | - |
| *T. ursinus* + *Trichuris* sp. + *T. suis* | - | -/79 | - | - | - | - |
