# Supplementary material for: Trichuris trichiura (Linnaeus, 1771) From Human and Non-human Primates: Morphology, Biometry, Host Specificity, Molecular Characterization, and Phylogeny
Source: Front Vet Sci. 2021 Feb 9;7:626120. doi: 10.3389/fvets.2020.626120 (PMC7934208; doi:10.3389/fvets.2020.626120)
Supplement: Supplementary file 4 [file Table_4.DOCX]

**Table S4.** Intra-specific and inter-specific similarity observed in concatenated ribosomal (ITS1 and ITS2) sequences in *Trichuris* species isolated from different host species. Hosts included in the clade 2: - (Subclade 2a, b, c) 1º: *C. aethiops*, *H. sapiens*, *N. leucogenys*, *P. anubis*, *P. hamadryas*, *P. h. ursinus, P. papio*, *R. roxellana*; 2º: *M. sylvanus, P. papio;* 3º: *M. leonine*, *M. sylvanus;* 4º: *C. aethiops*, *E. patas.*  –Subclade *Trichuris* sp. (*M. mulatta): M. mulatta*; -Subclado *Trichuris* sp. (*T. francoisi): T. francoisi*. Hosts included in the clade 1: -Subclade *T. suis*: *S. scrofa* , *S.s. domestica*; Subclade *T. colobae*: *C. g. kikuyensis*, *N. gabriellae*; Sublcade *T. ursinus*: *P. ursinus*; Subclade *T. trichiura* (Africa): *C. ascanius, C. guereza, C. lhoesti , C. mitis, H. sapiens, L. albigenal, P. rufomitratus, P. anubis, P. troglodytes.*

| CLADE 2 | *T. trichiura*  (Subclade 2a, b, c) 1º | *T. trichiura*  (Subclade 2a, b, c) 2º | *T. trichiura*  (Subclade 2a, b, c) 3º | *T. trichiura*  (Subclade 2a, b, c) 4º | *Trichuris* sp.  (Subclade *M. mulatta*) | *Trichuris* sp.  (Subclade *T. francoisi*) | *T. suis* | *T. colobae* | *T. ursinus* | *T. trichiura* (Africa) |
| --- | --- | --- | --- | --- | --- | --- | --- | --- | --- | --- |
| *T. trichiura* (Subclade 2a, b, c) 1º | 99.71-100 |  |  |  |  |  |  |  |  |  |
| *T. trichiura* (Subclade 2a, b, c) 2º | 97.99-98.91 | 98.04 |  |  |  |  |  |  |  |  |
| *T. trichiura* (Subclade 2a, b, c) 3º | 96.38-99.65 | 94.99-99.02 | 96.03-100 |  |  |  |  |  |  |  |
| *T. trichiura* (Subclade 2a, b, c) 4º | 97.41-99.42 | 96.49-98.79 | 94.65-99.65 | 97.24-98.68 |  |  |  |  |  |  |
| *Trichuris* sp. (Subclade *M. mulatta*) | 97.93-98.22 | 96.89-97.47 | 95.28-98.33 | 96.14-98.10 | 99.88 |  |  |  |  |  |
| *Trichuris* sp. (Subclade *T. francoisi*) | 95.68-95.97 | 94.82-95.11 | 93.21-95.97 | 93.73-95.74 | 95.97-96.09 | 99.77-99.94 |  |  |  |  |
| CLADE 1 |  |  |  |  |  |  |  |  |  |  |
| *T. suis* | 91.66-91.89 | 91.02-91.31 | 90.97-92.23 | 90.62-92.17 | 91.83-91.94 | 91.48-91.54 | 99.77 |  |  |  |
| *T. colobae* | 88.90-91.08 | 88.32-90.39 | 88.55-91.31 | 87.92-91.31 | 88.84-90.91 | 87.92-90.39 | 90.85-95.80 | 92.64 |  |  |
| *T. ursinus* | 91.02-91.14 | 90.33-90.74 | 90.33-91.48 | 90.10-91.37 | 91.25 | 91.14-91.20 | 96.72-96.66 | 90.74-96.03 | 100 |  |
| *T. trichiura* (Africa) | 90.91-91.14 | 90.45-90.79 | 90.28-91.48 | 90.16-91.37 | 91.13-91.25 | 90.79-90.97 | 96.61-96.84 | 91.14-96.09 | 98.79-98.96 | 99.83-100 |
