# Supplementary material for: Trichuris trichiura (Linnaeus, 1771) From Human and Non-human Primates: Morphology, Biometry, Host Specificity, Molecular Characterization, and Phylogeny
Source: Front Vet Sci. 2021 Feb 9;7:626120. doi: 10.3389/fvets.2020.626120 (PMC7934208; doi:10.3389/fvets.2020.626120)
Supplement: Supplementary file 5 [file Table_5.DOCX]

**Table S5.** Intra-specific and inter-specific similarity observed in *cox*1 partial sequences in *Trichuris* species isolated from different host species. Hosts included in the clade 2: -Subclade 2a: *H. sapiens*, *M. sylvanus*; -Subclade 2b: *H. sapiens*, *P. anubis*; -Subclade 2c: *C. aethiops*, *E. patas*, *H. sapiens*, *M. fuscata*, *M. sylvanus*, *P. hamadryas*, *P. papio*, *Papio* sp.; -Subclade 2d: *M. fuscata*.

|  | *T. trichiura* (Subclade 2a) | *T. trichiura* (Subclade 2b) | *T. trichiura.* (Subclade 2c) | *T. trichiura* (Subclade 2d) Subcl*. M. fuscata* | *T. suis* | *T. colobae* | *T. ursinus* | *Trichuris* sp. (*Chlorocebus*) |
| --- | --- | --- | --- | --- | --- | --- | --- | --- |
| *T. trichiura* (Subclade 2a) | 97.64-98.65 |  |  |  |  |  |  |  |
| *T. trichiura* (Subclade 2b) | 84.80-86.49 | 94.93-100 |  |  |  |  |  |  |
| *T. trichiura* (Subclade 2c) | 83.11-84.12 | 79.05-82.09 | 97.30-100 |  |  |  |  |  |
| *T. trichiura* (Subclade 2d) Subcl*. M. fuscata* | 84.46-86.49 | 82.09-83.45 | 85.14-88.18 | 97.64-99.32 |  |  |  |  |
| *T. suis* | 76.69-79.05 | 76.69-81.08 | 77.03-78.72 | 75-79.39 | 90.88-100 |  |  |  |
| *T. colobae* | 79.05-81.08 | 78.04-80.74 | 76.01-77.03 | 76.35-78.38 | 79.05-81.42 | 99.66-100 |  |  |
| *T. ursinus* | 77.36-78.38 | 72.97-76.01 | 75-76.35 | 75-75.68 | 81.42-84.46 | 81.76-82.09 | 100 |  |
| *Trichuris* sp. (*Chlorocebus*) | 78.72-80.41 | 74.32-75.68 | 77.03-78.72 | 77.03-78.72 | 79.39-81.76 | 80.74-81.76 | 82.09-82.77 | 96.62-100 |
