# Supplementary material for: Trichuris trichiura (Linnaeus, 1771) From Human and Non-human Primates: Morphology, Biometry, Host Specificity, Molecular Characterization, and Phylogeny
Source: Front Vet Sci. 2021 Feb 9;7:626120. doi: 10.3389/fvets.2020.626120 (PMC7934208; doi:10.3389/fvets.2020.626120)
Supplement: Supplementary file 6 [file Table_6.DOCX]

**Table S6.** Intra-specific and inter-specific similarity observed in *co*b partial sequences in *Trichuris* species isolated from different host species. Hosts included in the clade 2: -Subclade 2a: *M. sylvanus*; -Subclade 2b: *H. sapiens*, *P. anubis*; -Subclade 2c: *C. aethiops*, *E. patas*, *H. sapiens*, *M. fuscata*, *M. sylvanus*, *P. hamadryas*, *P. papio*, *Papio* sp.; -Subclade 2d: *M. fuscata*.

|  | *T. trichiura* (Subclade 2a) | *T. trichiura* (Subclade 2b) | *T. trichiura* (Subclade 2c) | *T. trichiura* (Subclade 2d) Subcl. *M. fuscata* | *T. suis* | *T. colobae* | *T. ursinus* | *Trichuris* sp. (*Chlorocebus)* |
| --- | --- | --- | --- | --- | --- | --- | --- | --- |
| *T. trichiura* (Subclade 2a) | 97.07-99.55 |  |  |  |  |  |  |  |
| *T. trichiura* (Subclade 2b) | 87.39-88.51 | 93.92-100 |  |  |  |  |  |  |
| *T. trichiura* (Subclade 2c) | 79.05-85.14 | 79.50-85.36 | 92.12-100 |  |  |  |  |  |
| *T. trichiura* (Subclade 2d) Subcl. *M. fuscata* | 84.46-86.49 | 85.36-87.61 | 82.43-89.19 | 98.65-100 |  |  |  |  |
| *T. suis* | 73.42-75.23 | 74.10-75.90 | 68.47-75.45 | 73.20-75.23 | 90.32-100 |  |  |  |
| *T. colobae* | 71.62-72.52 | 72.97 | 69.82-74.10 | 73.42-73.87 | 77.48-79.05 | 100 |  |  |
| *T. ursinus* | 72.97-73.42 | 73.87-74.55 | 68.47-74.55 | 74.10-74.77 | 76.35-78.60 | 78.15-78.60 | 99.10-99.77 |  |
| *Trichuris* sp. (*Chlorocebus)* | 71.62-73.20 | 73.65-74.77 | 68.47-74.55 | 73.20-75.22 | 75.90-79.05 | 75.23-76.35 | 79.28-80.63 | 93.69-100 |
