# Supplementary material for: Trichuris trichiura (Linnaeus, 1771) From Human and Non-human Primates: Morphology, Biometry, Host Specificity, Molecular Characterization, and Phylogeny
Source: Front Vet Sci. 2021 Feb 9;7:626120. doi: 10.3389/fvets.2020.626120 (PMC7934208; doi:10.3389/fvets.2020.626120)
Supplement: Supplementary file 7 [file Table_7.DOCX]

**Table S7.** Intra-specific and inter-specific similarity observed in *rrn*L partial sequences in *Trichuris* species isolated from different host species. Hosts included in the clade 2: -Subclade 2a: *M. sylvanus*; -Subclade 2b: *H. sapiens*, *P. anubis*; -Subclade 2c: *C. sabaeus*, *H. sapiens*, *M. fuscata*, *M. sylvanus*, *P. hamadryas*, *P. papio*, *Papio* sp.; -Subclade 2d: *M. fuscata*; -Subclado *Trichuris* sp.: *T. francoisi*.

|  | *T. trichiura* (Subclade 2a) | *T. trichiura* (Subclade 2b) | *T. trichiura* (Subclade 2c) | *T. trichiura* (Subclade 2d) Subcl*. M. fuscata* | *Trichuris* sp. (Subclade *T. francoisi*) | *T. suis* | *T. colobae* | *Trichuris* sp. (*Chlorocebus*) |
| --- | --- | --- | --- | --- | --- | --- | --- | --- |
| *T. trichiura* (Subclade 2a) | 98.88 |  |  |  |  |  |  |  |
| *T. trichiura* (Subclade 2b) | 94.69-95.25 | 98.04-100 |  |  |  |  |  |  |
| *T. trichiura* (Subclade 2c) | 91.34-93.02 | 91.62-93.58 | 97.77-100 |  |  |  |  |  |
| *T. trichiura* (Subclade 2d) Subcl. *M. fuscata* | 93.85-94.97 | 95.53-96.93 | 93.29-95.53 | 98.88-100 |  |  |  |  |
| *Trichuris* sp. (Subclade *T. francoisi*) | 84.08-84.64 | 83.80-84.92 | 85.20-86.31 | 85.75-86.31 | 98.88-100 |  |  |  |
| *T. suis* | 79.05-79.89 | 80.73-82.12 | 80.17-82.12 | 81.56-82.68 | 80.73-82.12 | 98.04-100 |  |  |
| *T. colobae* | 80.17-81.28 | 79.61-82.12 | 81.84-87.71 | 81.01-82.68 | 79.33-80.73 | 85.75-87.71 | 92.74-100 |  |
| *Trichuris* sp. (*Chlorocebus*) | 80.17-82.12 | 80.45-82.96 | 82.12-84.36 | 81.01-83.24 | 80.17-80.73 | 86.03-87.15 | 86.59-88.55 | 97.49-99.72 |
