# Supplementary figures and images for: Trichuris trichiura (Linnaeus, 1771) From Human and Non-human Primates: Morphology, Biometry, Host Specificity, Molecular Characterization, and Phylogeny
Source: Front Vet Sci. 2021 Feb 9;7:626120. doi: 10.3389/fvets.2020.626120 (PMC7934208; doi:10.3389/fvets.2020.626120)

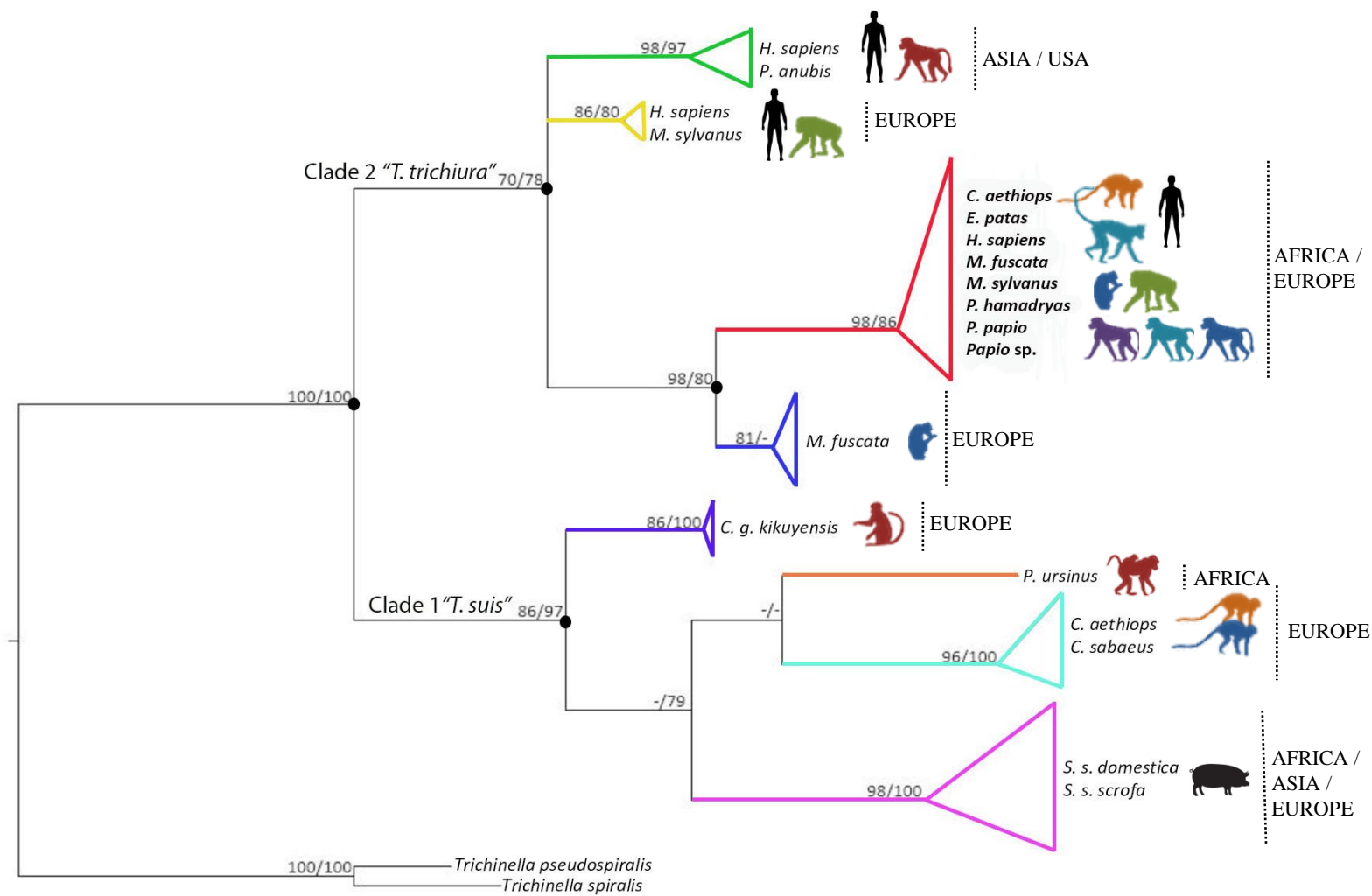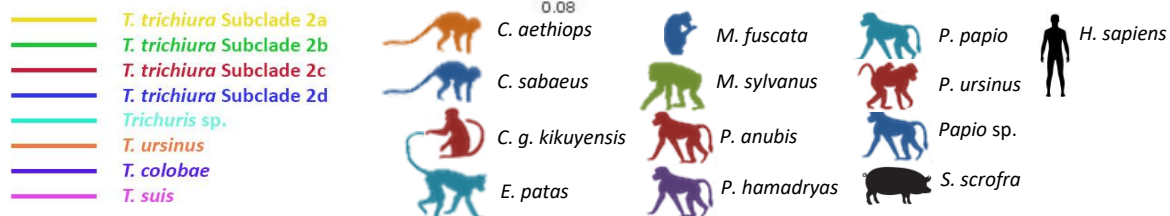

Supplement: Supplementary file 8 [file Data_Sheet_1.PDF]

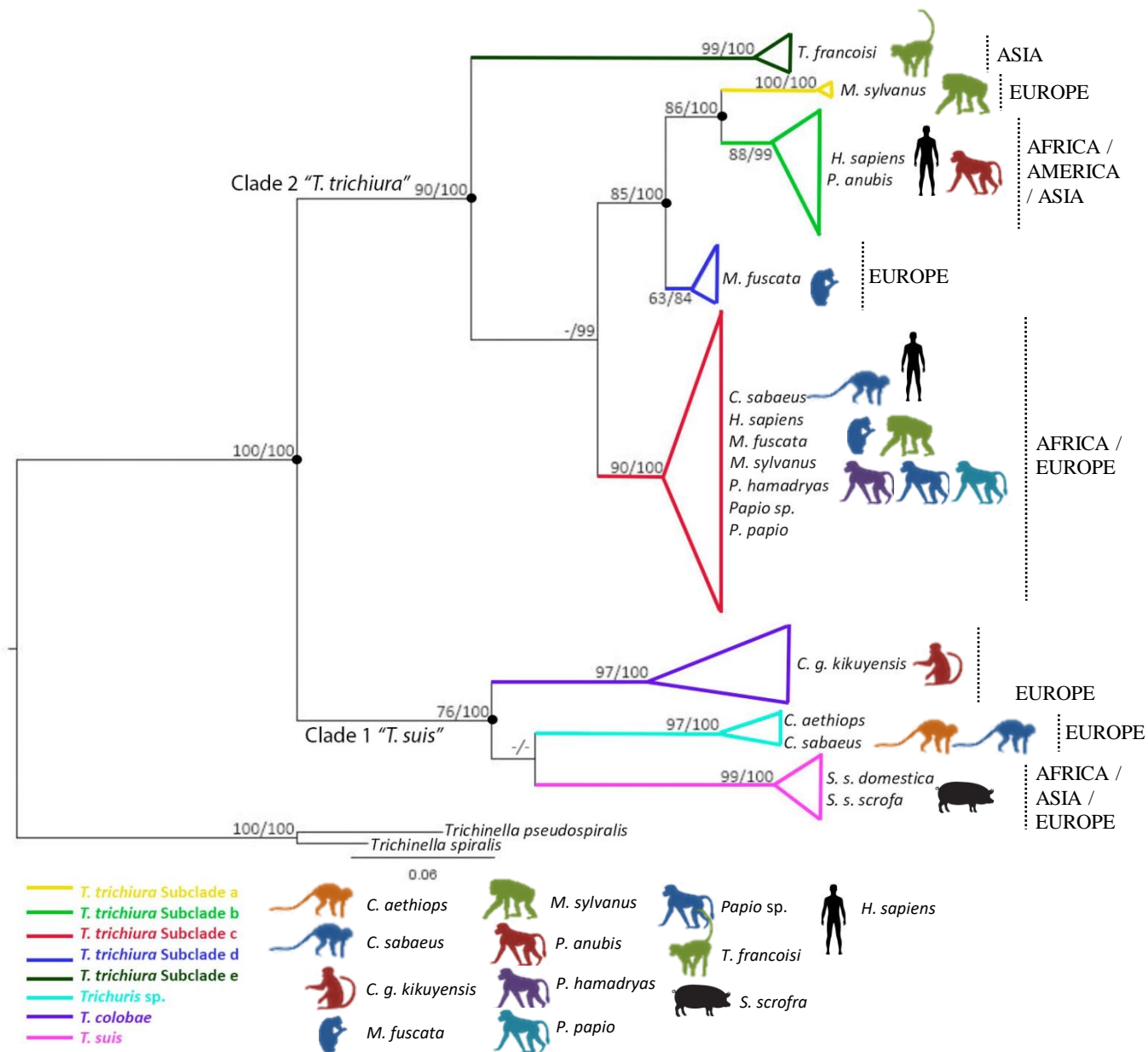

Supplement: Supplementary file 10 [file Data_Sheet_3.PDF]
