# Supplementary material for: Trichuris trichiura (Linnaeus, 1771) From Human and Non-human Primates: Morphology, Biometry, Host Specificity, Molecular Characterization, and Phylogeny
Source: Front Vet Sci. 2021 Feb 9;7:626120. doi: 10.3389/fvets.2020.626120 (PMC7934208; doi:10.3389/fvets.2020.626120)
Supplement: Supplementary file 9 [file Data_Sheet_2.PDF]

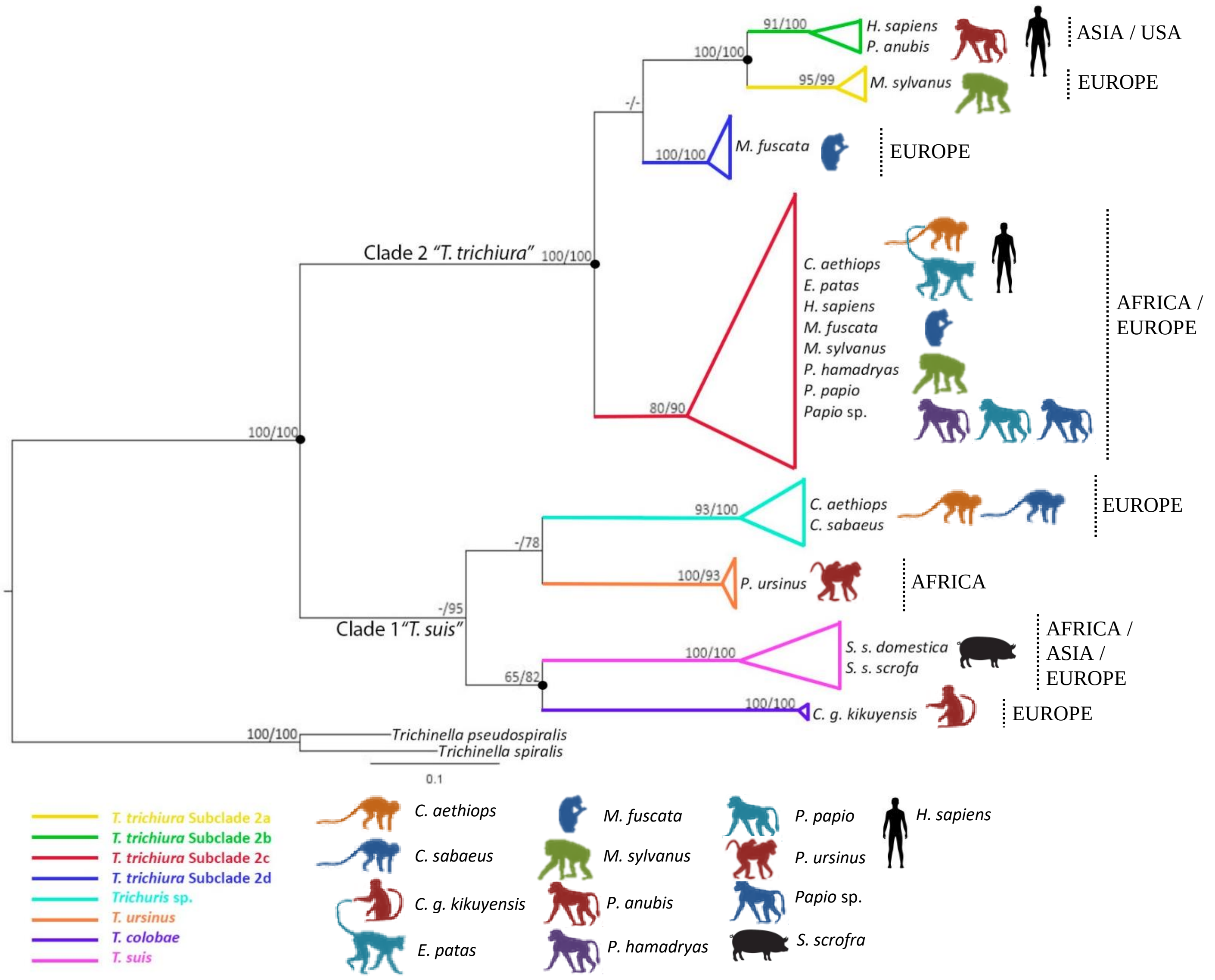
91/100
H. sapiens
P. anubis
ASIA / USA
100/100
95/99
M. sylvanus
EUROPE
-/-
100/100
M. fuscata
EUROPE
Clade 2 "T. trichiura"
100/100
C. aethiops
E. patas
H. sapiens
M. fuscata
M. sylvanus
P. hamadryas
P. papio
Papio sp.
AFRICA / EUROPE
80/90
100/100
93/100
C. aethiops
C. sabaeus
EUROPE
-/78
100/93
P. ursinus
AFRICA
-/95
Clade 1 "T. suis"
100/100
S. s. domestica
S. s. scrofa
AFRICA / ASIA / EUROPE
65/82
100/100
C. g. kikuyensis
EUROPE
100/100
Trichinella pseudospiralis
Trichinella spiralis
0.1
T. trichiura Subclade 2a
T. trichiura Subclade 2b
T. trichiura Subclade 2c
T. trichiura Subclade 2d
Trichuris sp.
T. ursinus
T. colobae
T. suis
C. aethiops
C. sabaeus
C. g. kikuyensis
E. patas
M. fuscata
M. sylvanus
P. anubis
P. hamadryas
P. papio
P. ursinus
Papio sp.
S. scrofra
H. sapiens
